# Cell therapy for Duchenne muscular dystrophy: promises, challenges, and controversies

**DOI:** 10.1007/s00018-025-05904-5

**Published:** 2025-10-11

**Authors:** Agnieszka Łoboda, Józef Dulak

**Affiliations:** Department of Medical Biotechnology, Faculty of Biochemistry, Biophysics and Biotechnology, Gronostajowa 7, Jagiellonian University in Kraków, Kraków, 30-387 Poland

**Keywords:** Cardiomyopathy, DMD, Induced pluripotent stem cells, Myoblasts, Satellite cells

## Abstract

Despite extensive studies, Duchenne muscular dystrophy, a neuromuscular disorder caused by the lack of dystrophin, a key muscle structural protein, remains an incurable disease. One of the potential treatment options currently being investigated is cell therapy, although it has not yet been clinically established. Several strategies, including muscle satellite cells, mesoangioblasts (vessel-associated multipotent stem cells), and induced pluripotent stem cell (iPSC)-derived muscle cells, have emerged as tools for restoring dystrophin expression and regenerating damaged muscle tissue. Nevertheless, each of these approaches faces significant limitations, including poor cell engraftment, low delivery efficiency, and the risk of immune rejection. Furthermore, long-term safety, the possibility of tumorigenicity, and off-target effects must be rigorously evaluated. Importantly, the latter technology, utilizing cardiomyocytes differentiated from iPSC, holds the potential for addressing cardiomyopathy, the major cause of death of DMD patients. At the same time, several interventions using cells with claimed stem cell potential have emerged, raising both scientific and ethical concerns. This review summarizes recent advancements in the development of cell therapies for DMD, highlighting promising progress while critically analysing questionable approaches.

## Introduction

Duchenne muscular dystrophy (DMD) represents one of the rare genetic disorders, affecting approximately 1 in 5,000–6,000 boys. The primary cause of the disease, which drives its rapid and irreversible progression, is the absence of functional dystrophin, a 427 kDa actin-binding protein and a crucial component of the dystrophin-glycoprotein complex (DGC). More than 7,000 patient-specific mutations have been identified in the *DMD* gene, the largest human gene, which contains 79 exons and approximately 2.4 million base pairs [1].

The predominant muscle phenotype of DMD is attributed to the absence of dystrophin, which connects the intracellular cytoskeleton to the extracellular matrix by linking F-actin and the DGC complex, functioning as the ‘shock absorber’ and force transducer in muscle and cardiomyocytes [2, 3]. DGC maintains the integrity of muscle fibers and regulates several cellular pathways, including nitric oxide (NO) synthesis and calcium entry [4, 5]. Therefore, a lack of dystrophin results in increased sarcolemmal damage caused by contractions and disrupts the homeostasis of NO and Ca^2+^. However, dystrophin appears to be also expressed in other cells, including cardiomyocytes, smooth muscle cells, and many non-muscle cells [6–9], although the presence and significance in fibroblasts and endothelial cells are disputable. Dystrophin was also claimed to serve roles beyond its structural function, e.g., it is presumed to be a tumor suppressor gene [10], though this hypothesis requires further validation. Accordingly, mutations in DMD patients, which either result in a complete loss of dystrophin or, in rarer cases, the production of dysfunctional protein, significantly affect the function of skeletal and respiratory muscles, the heart, the nervous system, and other organs (for a review, see e.g [11]).

DMD patients suffer from progressive muscle dystrophy with a plethora of clinical signs. The first symptoms of the disease, identified mostly by parents, are frequently noticeable in 1-3-year-old boys and manifest, among others, as a struggle to sit down, stand up, walk, jump, climb stairs, and run. In young boys, symptoms often include calf muscle enlargement, poor head control, muscle pain, and general clumsiness. A characteristic feature is the Gowers’ sign, where weakness in the proximal muscles forces the child to use their hands to rise from a lying position.

**Fig. 1:**
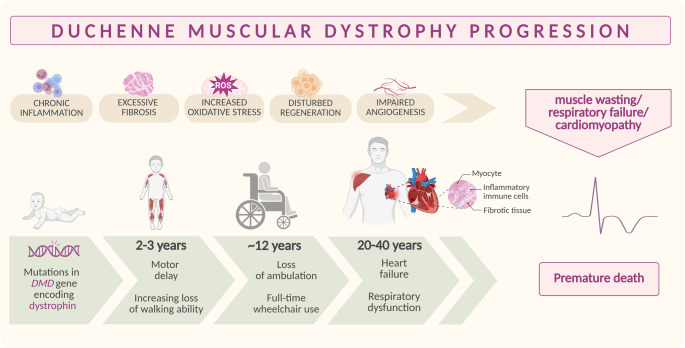
DMD progression. Mutations in the *DMD* gene, resulting in the absence of dystrophin, lead to muscle degeneration and heart complications. These are exacerbated by chronic inflammation, accelerated fibrosis, increased oxidative stress, disturbed regeneration, and impaired angiogenesis. Symptoms typically begin in early childhood (2–3 years), with weakness in the lower limbs, difficulty walking, and gait abnormalities. In adolescence (~ 12 years), most individuals are wheelchair-bound, and respiratory and cardiac issues become more severe. In adulthood, ventilatory support is often required, and life expectancy is significantly reduced due to respiratory and heart failure

Between the ages of 8 and 14, patients lose ambulation due to gradual muscle atrophy and become dependent on wheelchairs. Scoliosis and joint contractures develop as muscular weakness increases, which in turn worsens restrictive lung disease [12]. Therefore, with disease progression, respiratory failure occurs, which can be alleviated through the use of nocturnal non-invasive ventilation (NIV), diurnal non-invasive ventilation *via* a mouthpiece, or, finally, ventilation *via* tracheostomy [13]. Furthermore, immobility increases the risk of fracture and accelerates the loss of bone density [14]. Of note, cardiorespiratory complications have become one of the primary causes of mortality from the second decade of life in DMD men. Importantly, female carriers are also at risk of cardiomyopathy [15–18]. Unfortunately, heart problems in DMD patients may be undetected without detailed examination [19, 20]. Many teenage individuals have no classic symptoms of heart failure, or these symptoms are not recognized properly, as they mostly use wheelchairs and do not undergo increased cardiac workload. This results in a significant delay in their proper assessment and the late start of pharmacological therapies [21]. Therefore, to prevent the early onset of heart failure, initiating cardiac-focused interventions before ventricular dysfunction is detected may be crucial. Nevertheless, so far there is no effective treatment for dystrophin-dependent cardiomyopathy, which is ultimately fatal in DMD patients (who, due to their very severe overall condition, typically do not qualify for heart transplantation), highlighting the need to identify novel therapeutic avenues and mechanistic insights [22–24]. In addition to well-known skeletal and cardiac muscle symptoms, DMD is associated with cognitive disorders, language, and learning disabilities, failure to thrive, and metabolic disorders (obesity, insulin resistance, glucose intolerance) [22, 25]. A summary of the disease progression and its key hallmarks is presented in Fig. 1.

## Satellite cell dysfunction in Duchenne muscular dystrophy

**Fig. 2:**
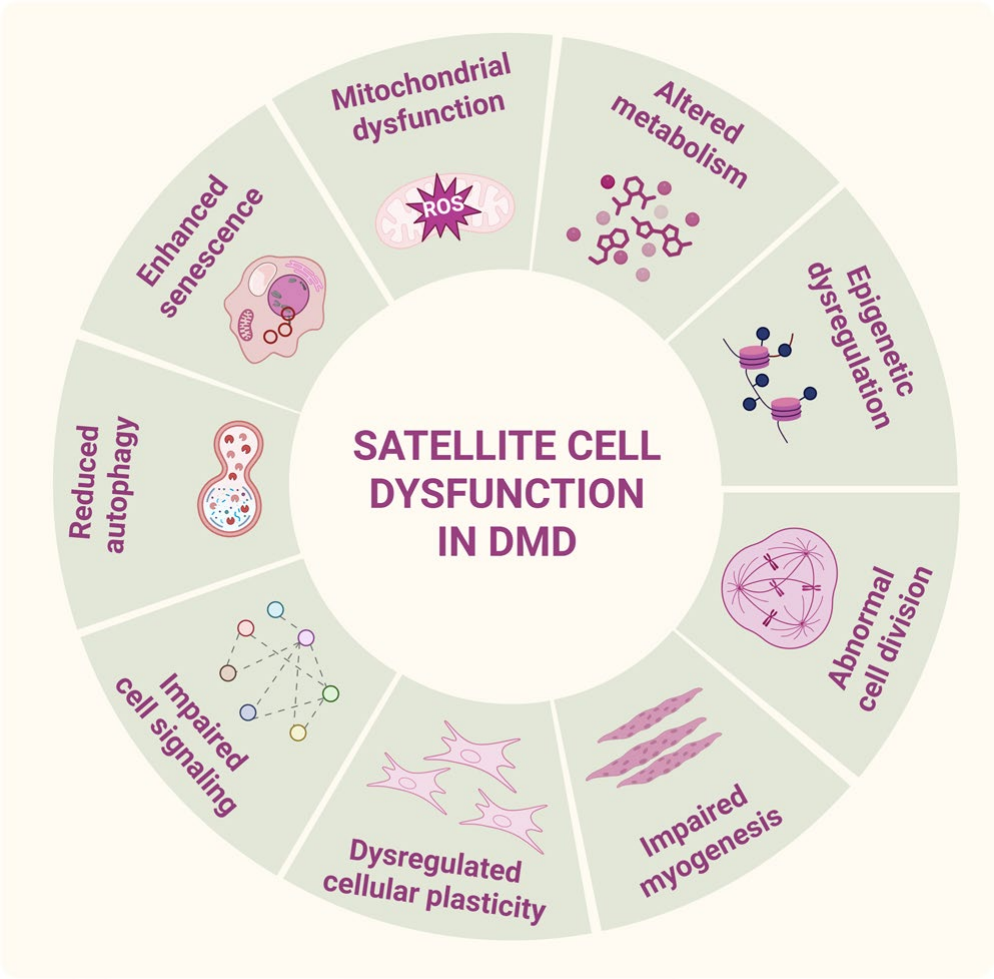
Satellite cell dysfunction in DMD. Dystrophic satellite cells exhibit a range of dysfunctions, including reduced asymmetric division, increased senescence, disrupted autophagy, impaired epigenetic regulation of gene expression, altered cell signaling and metabolism, and mitochondrial dysfunction. Dystrophin deficiency also leads to satellite cells’ impaired differentiation and dysregulated plasticity, ultimately promoting a fibrotic rather than myogenic lineage commitment

In DMD, impaired muscle regeneration is linked to disturbed production of myogenic progenitor cells. Importantly, dystrophin is not only a structural protein in muscle fibers but is also present in muscle stem cells, known as satellite cells (mSCs), characterized as CD45⁻CD31⁻α7-integrin⁺Sca-1⁻ cells that express Pax7, a key marker essential for their survival and proper function [26]. Dumont et al. [27] demonstrated that dystrophin plays a critical role in regulating the polarity and asymmetric division of mSCs. In regenerating healthy muscle, activated mSCs undergo both symmetric cell division to maintain the muscle stem cell pool and asymmetric cell division to give rise to myogenic progenitor cells that expand and contribute to efficient muscle repair and regeneration. In dystrophic skeletal muscle cells, symmetric expansion dominates over asymmetric cell division, resulting in a reduced pool of myogenic progenitor cells and impaired muscle regeneration. Interestingly, although altered, both increased [28–31] and decreased [32, 33] SC numbers have been reported in various animal dystrophic models; their functional exhaustion due to telomere shortening, induction of cellular senescence, and suppression of proliferation has been consistently observed [34, 35]. Our studies performed in *mdx* mice (the commonly used murine DMD model with a point mutation in exon 23 of the *Dmd* gene) suggest that the compromised regenerative potential is not primarily due to a decreased abundance of mSCs, as we found a higher number of Pax7^+^ cells in dystrophic muscles [36–38]. However, in D2-*mdx* mice, which demonstrate more severe muscle damage, the number of satellite cells is lower than in healthy DBA counterparts [32]. Moreover, several studies, including ours, indicated that murine mSCs isolated from *mdx* mice [39] or human embryonic stem cell (hESC)-derived mSCs, as reported by Chal et al. [40], present disturbed differentiation. Additional pathological features of dystrophic mSCs include mitochondrial abnormalities evidenced by atypical morphology [41, 42], altered mitochondrial gene expression, and significantly diminished respiratory function [43]. Granet et al. [32] found reduced expression of autophagy-related genes in mSCs isolated from *mdx* and D2-*mdx* mice, and demonstrated that treatment with an autophagy-promoting peptide enhanced the differentiation capacity of DMD progenitor cells. Notably, analysis of single-nuclei human DMD transcriptomic data also revealed dysregulated autophagy pathways in diseased mSCs [32], underscoring the universal role of impaired autophagy in their dysfunction. All of these defects may result in elevated oxidative stress, higher production of reactive oxygen species (ROS), reduced ATP generation, and changes in overall cellular metabolism. Notably, dystrophin restoration using CRISPR/Cas9 editing has been shown to improve the energetic properties of dystrophic progenitor cells [44]. In addition, DMD pathology is associated with the premature onset of senescence of mSCs, which may contribute to their impaired regenerative capacity [45, 46]. Moreover, dystrophic mSCs, due to elevated transforming growth factor beta (TGFβ) signaling, may display enhanced fibrogenic activity at the expense of myogenic potential [47]. Such misguided differentiation reflects changes in cellular plasticity, resulting in excessive connective tissue formation and a more pro-fibrotic environment in affected muscles. Finally, as mSCs rely on tightly regulated epigenetic mechanisms and signaling pathways to maintain optimal function, the lack of dystrophin may disrupt these regulatory networks, resulting in regenerative failure of dystrophic muscle. It should be emphasized, however, that the primary cause of the disease is the absence of dystrophin in muscles, which leads to fiber degeneration. Satellite cell dysfunction, described here briefly, summarized in Fig. 2, and comprehensively discussed in several recent review papers [48, 49], may contribute to disease progression over time.

Taking all factors into consideration, restoring the proper function of mSCs appears to be crucial. Some studies suggest that treatment with various pharmacological compounds that modify signaling pathways may, for example, correct impaired mSC polarity (for references, see [48, 49]). However, a key unresolved technical question regarding potential DMD treatments remains the effectiveness of gene therapy, specifically, whether mSCs can be successfully transduced, and if so, what the long-term impact of such transduction is on their function.

### Treatment of Duchenne muscular dystrophy

**Fig. 3:**
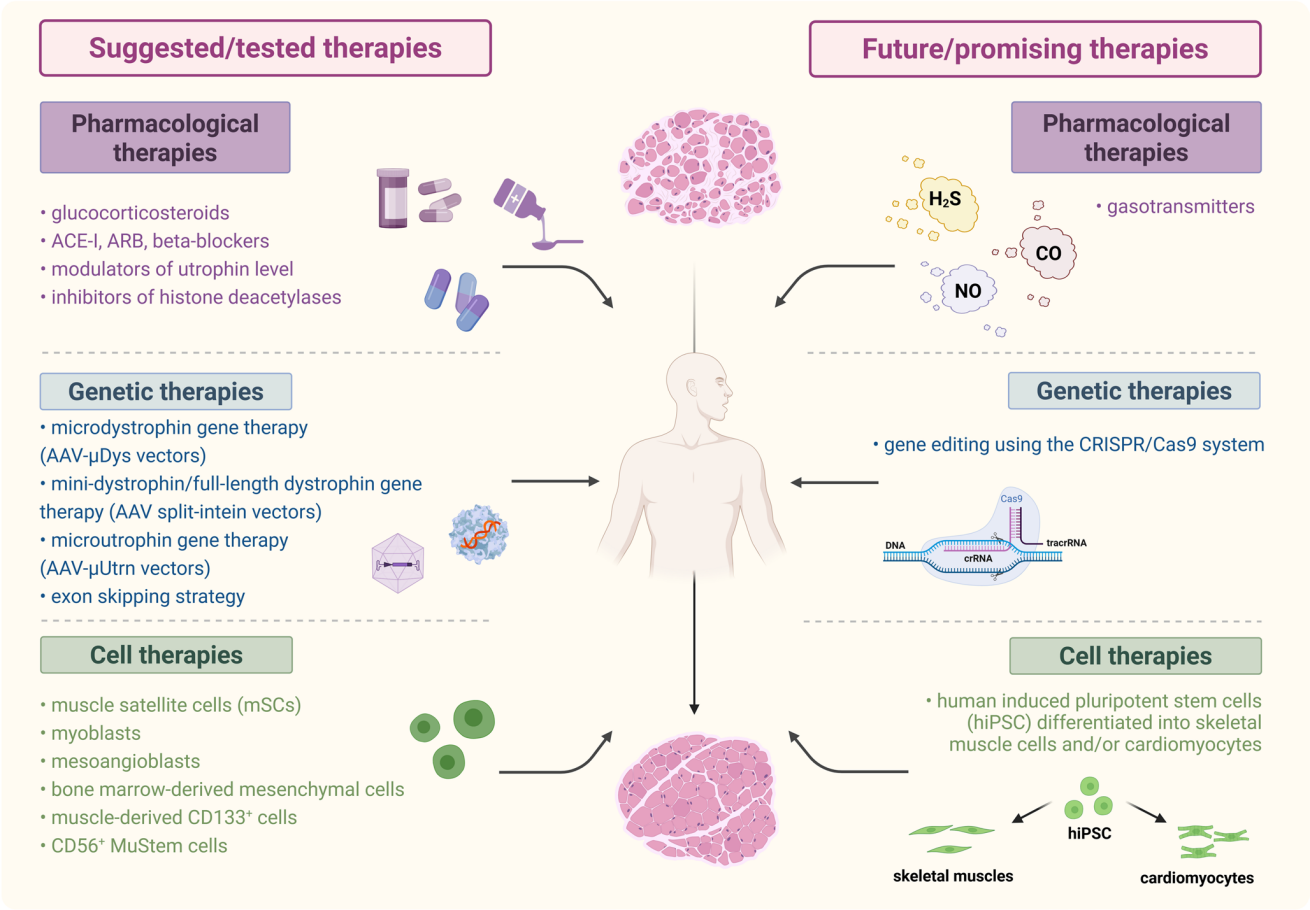
Treatment of DMD. The key treatment strategies employed to manage the disease include pharmacological therapies, mostly glucocorticosteroid treatment to slow muscle degeneration; genetic therapies to restore micro-, mini-, or full-length dystrophin and microutrophin; and cell therapies with muscle satellite cells, myoblasts, and mesoangioblasts to regenerate muscle tissue. Among these strategies, future therapies are focused on the use of therapeutic gasotransmitters, like hydrogen sulfide, the CRISPR/Cas9 system for gene editing, and induced pluripotent stem cell-derived muscle cells or cardiomyocytes for cell-based approaches

Despite enormous efforts in basic research and a better understanding of the molecular mechanisms, effective therapies for DMD remain elusive. Current therapeutic strategies can be divided into three approaches (Fig. 3). The commonly used pharmacological treatment (Table 1, with detailed references) only mitigates the downstream effects of dystrophin deficiency, like inflammation, fibrosis, and oxidative stress. The gold standard drugs, used for more than 30 years, glucocorticoids (prednisone, prednisolone, and deflazacort), through reducing inflammatory response by inhibition of the NK-κB pathway, can prolong the ambulation of patients, increase muscle strength, slow the progression of scoliosis, and improve cardiovascular functions [50–53]. Despite these undeniable benefits on cardiac and skeletal muscle performance, their long-term daily use is associated with a high risk of several side effects, including weight gain, delayed growth, osteoporosis, adrenal insufficiency, behavioral changes, and gastrointestinal complications [54]. Some studies suggest that administering glucocorticoids less frequently, such as twice a week instead of daily, may be equally effective while causing fewer adverse effects [52, 55]. More recently, vamorolone (VBP-15), a synthetic anti-inflammatory steroid and mineralocorticoid receptor antagonist, has been shown to offer similar benefits with a more favorable side-effect profile. In vivo studies on *mdx* dystrophic mice, along with clinical trials, have demonstrated a positive effect from vamorolone administration [54, 56]. Given the encouraging results from Phase 1 and 2 trials, the Food and Drug Administration (FDA) registered this drug in October 2023, followed by approval from the European Medicines Agency (EMA) in December 2023. The Phase 3 VISION-DMD trial (NCT03439670, ClinicalTrials.gov) further confirmed the drug’s efficacy, as well as its favorable safety and tolerability profile over a 48-week treatment period. Moreover, cardiac abnormalities are, at least to some extent, delayed by treatment with angiotensin-converting enzyme inhibitors (ACEi), aldosterone receptor blockers (ARBs), β-adrenergic receptor (β-AR) blockers, and mineralocorticoid receptor antagonists (MRAs). Also, modulators of utrophin (the paralogue of dystrophin) or inhibitors of histone deacetylases are being tested (reviewed in [11]). The ongoing research also explores the therapeutic potential of gasotransmitters, including hydrogen sulfide (H_2_S) [57–61], in mitigating disease progression (see Table 1 for additional information and references).

**Table 1:** Examples of Pharmacological DMD therapies, used in patients or tested in animal models and/or in humans. Drugs officially approved and registered for the treatment of DMD are indicated in bold

Type of drug/compound	Exemplary drug/compound	Mechanism of action	References
glucocorticoids	prednisone prednisolone **deflazacort**	inhibition of the NK-κB pathway and reducing the inflammatory response	[50–53]
synthetic steroids	**vamorolone**	anti-inflammatory; fewer side effects due to selective mineralocorticoid receptor antagonism	[54, 56]
PDE5 inhibitors	sildenafil tadalafil	inhibiting of cGMP phosphodiesterase (PDE5) activity and prolongation of the biological half-life of cGMP	[52, 170]
ACEi angiotensin-converting enzyme inhibitors	perindopril enalapril captopril	inhibition of Ang II formation and metabolism	[21, 171–173]
ARBs angiotensin receptor blockers	losartan	competitive inhibition of angiotensin II (Ang II); binding to the angiotensin 1 receptor	[21, 174, 175]
β-AR beta-adrenergic receptor	bisoprolol metoprolol carvedilol	inhibition of β-adrenergic receptor (selective or nonselective)	[176–178]
MRAs mineralocorticoid receptor antagonists	eplerenone spironolactone	blocking the endogenous mineralocorticoid, aldosterone, at its receptors	[179–181]
utrophin upregulation	ezutromid	an aryl hydrocarbon receptor (AhR) antagonist; failed in clinical trials	[182, 183]
HDACi histone deacetylase inhibitors	**givinostat** suberoylanilide hydroxamic acid (SAHA)	inhibition of histone deacetylase activity	[184]
H _2_S donors	sodium hydrosulfide (NaHS) sodium sulfide (Na_2_S) GYY4137	an increase in hydrogen sulfide levels results in anti-inflammatory, antioxidant, anti-fibrotic, and proangiogenic effects (tested in animal DMD models)	[57, 61, 185–187]

Although pharmacological therapies can improve the quality of life of DMD patients and slow disease progression, they do not target the underlying cause of the disorder. In contrast, this could be achieved with the use of genetic therapies, such as read-through therapy, an antisense oligonucleotide (ASO)-mediated exon skipping, vector-mediated gene therapy to restore expression of a shorter form of dystrophin (micro- or mini-dystrophin), or microutrophin and utrophin, the paralogue of dystrophin, expressed during fetal development, and then largely replaced by dystrophin in mature muscle fibers [62, 63], and CRISPR/Cas9-based gene editing (Fig. 3) [64, 65]. It has to be underlined that delivery of the full-length dystrophin gene is challenging because of the large size of its cDNA (~ 11.2 kb protein-coding sequence [66, 67]), which prevents its packaging to adeno-associated viral (AAV) vectors commonly used for muscle cell transduction, able to carry only ~ 4.7 kb transgene [68]. However, innovative methods based on the split-intein technique have recently offered a new way to overcome such limitations and produce full-length dystrophin [69–71].

Finally, cell-based therapies may present a promising avenue for treating DMD (Fig. 3). This approach involves delivering cells capable of expressing dystrophin and could be considered a form of gene therapy, where the cell itself serves as the vector. However, expectations for cell therapy extend beyond gene replacement, as these cells are anticipated not only to express dystrophin but also to differentiate into functional muscle fibers, thereby fully restoring muscle integrity.

## Cell therapies for Duchenne muscular dystrophy

Over the years, various cell types have been considered for DMD cell therapy, including some that lack muscle-regenerative potential. However, given the progressive muscle degeneration characteristic of DMD, only cells capable of differentiating into skeletal muscle have the potential to provide meaningful therapeutic benefits.

As mentioned above, the CD45^−^CD31^−^α7-integrin^+^Sca-1^−^ mSCs are *bona fide* muscle stem cells that differentiate into myoblasts and ultimately form muscle fibers (for a review and references, see [72]). These cells, characterized by the expression of the transcription factor Pax7 and other markers, depending on the state of myogenic activity, are naturally present in skeletal muscles and the diaphragm, making them an obvious candidate for DMD cell therapy [73]. However, dystrophic satellite cells already have impaired functions ([27]; for references see [48, 49]), and when differentiated into muscle, they would not express dystrophin. Accordingly, satellite cell-based therapy requires either the use of allogeneic cells or genetic modification of the patient’s satellite cells to re-express dystrophin.

**Fig. 4:**
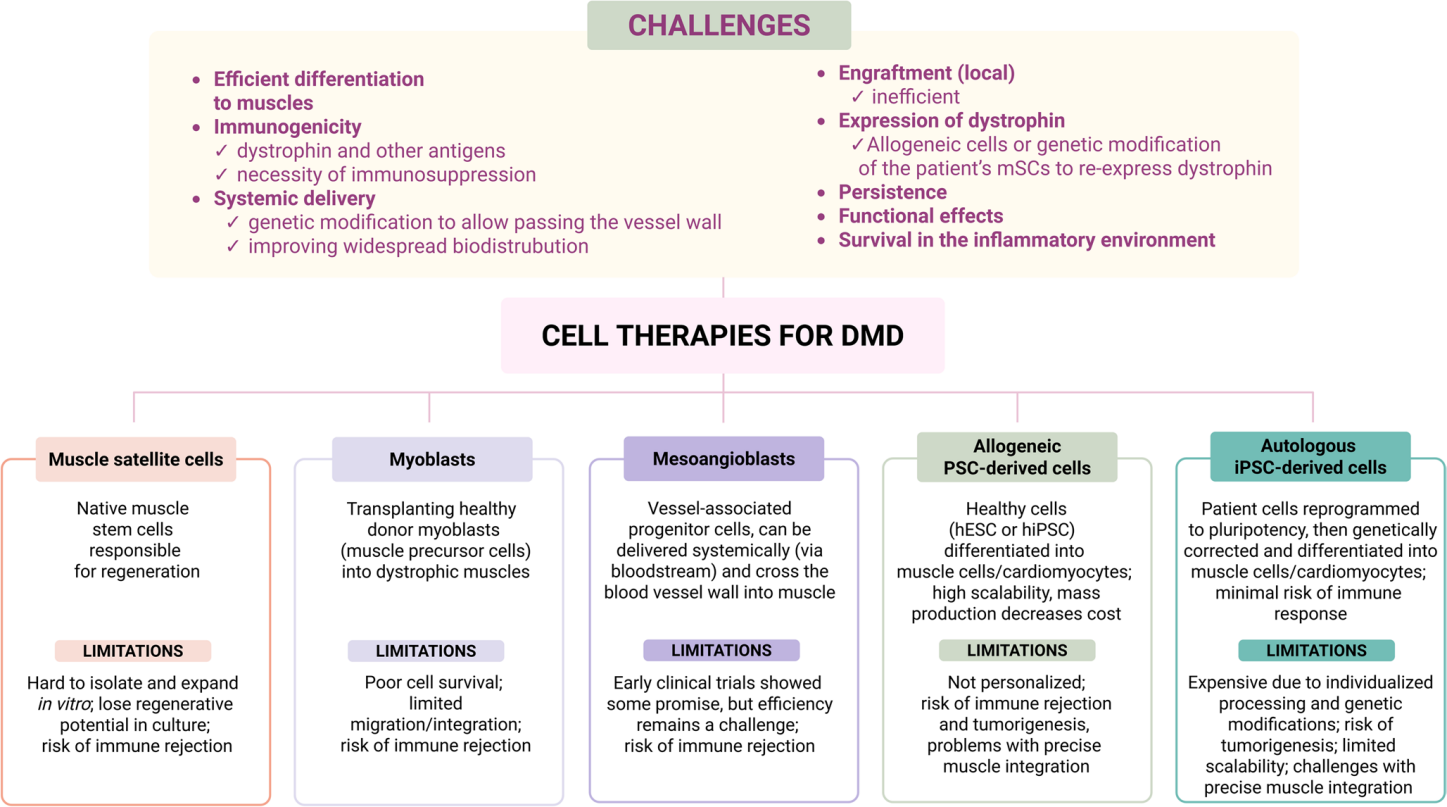
Challenges and promises of cell therapies for DMD. Although cell therapies hold considerable promise, they are associated with numerous challenges (as indicated in the upper part of the figure). Among the various well-justified approaches, the use of muscle satellite cells, myoblasts, mesoangioblasts, and pluripotent stem cell-derived muscle cells and cardiomyocytes is accompanied by serious limitations (depicted in the lower part of the figure and discussed in the text)

Cell therapy for DMD must overcome several barriers (Fig. 4). First, isolating and expanding a sufficient number of undifferentiated muscle progenitors capable of repopulating all damaged muscles remains difficult. Second, in the case of the allogeneic approach, overcoming genetic differences between the donor cells and the patient requires immunosuppression. Third, for autologous transplantation, the restoration of dystrophin expression in mSCs of DMD patients would be necessary. However, fourth, the potential immune response against dystrophin has to be carefully considered. Fifth, efficient cell therapy must ensure the high and long-term engraftment of cells across multiple muscles, enabling not only fiber regeneration but also the establishment of a functional satellite cell pool to support future muscle repair. To achieve widespread cell engraftment, systemic delivery appears to be the most logical strategy. Nevertheless, a major limitation is that myoblasts are unable to cross the blood vessel barrier (for a review, see [72]). While local injection into the muscle was tested and demonstrated to be feasible, it only allows for the correction of limited areas within small, superficial muscles [74].

### Satellite cell-based approaches

Transplantation of healthy mSCs has been tested in animal models of muscle injury, demonstrating the feasibility of this approach [75–80]. However, these experiments have also highlighted several limitations. First, the isolation of human mSCs requires an invasive muscle biopsy. Second, the long-term culture of such mSCs results in the loss of their regenerative potential, as the cells undergo senescence [77, 78, 81, 82]. Third, the mSCs isolated from healthy donors are immunologically mismatched with potential recipients, raising concerns about immune rejection. Nevertheless, animal studies have demonstrated the feasibility of their intramuscular application for muscle progenitor/stem cell therapy [83]. The fusion of healthy myoblasts with dystrophin-deficient fibers in *mdx* mice has been demonstrated [84], generating hope for a similar effect in patients [85]. While the initial, nonrandomized trial of myoblast transfer for DMD therapy claimed some positive effects [86, 87], the overall results were negative [88], indicating a lack of long-term myoblast engraftment and minimal dystrophin expression in the injected muscles [89–91]. Despite being administered in large numbers (exceeding 100 million cells) and through multiple injections, myoblasts failed to engraft effectively due to immune rejection (in the absence of immunosuppression), limited migration beyond the injection site, and their differentiated state.

Intravenous delivery of various stem cells, including mSCs/myoblasts, is ineffective, as the cells become trapped in the lungs and fail to cross the blood vessel wall to reach the muscles [92]. The intra-arterial injection has thus been tested as an alternative; however, its efficacy remains low, with only about 3.2% of injected cells detected in the analyzed muscle Sect. [93]. In the latter study performed in macaques, intra-arterial delivery of β-galactosidase-labeled myoblasts resulted in cell retention in vessels and inefficient muscle penetration. Moreover, even when cells are delivered intramuscularly, they do not survive well (for a review, see [94]). Recently, Saleh et al. [95] demonstrated that human skeletal muscle progenitor cells (isolated from 18-week human fetal muscle) cannot be efficiently systemically delivered into the muscle of immunodeficient dystrophic mice. Importantly, the intravenous delivery was ineffective, while intra-arterial administration allowed limited myoblast extravasation into muscle tissue; however, the efficacy was too low to achieve robust long-term engraftment. The cells have been mainly detected inside blood vessels, and the prominent clotting of these cells was noted in the dystrophic *mdx*D2-NSG (immunodeficient, to prevent rejection) mice [95]. This again indicates that the systemic delivery of myoblasts, as proposed by some approaches, is unjustified, in light of this and many previous studies.

### Mesoangioblasts and similar cells

Pericytes associated with vascular walls in the human skeletal muscle have been demonstrated to differentiate into muscle fibers when injected into murine skeletal muscles. These vessel-associated progenitors are referred to as mesoangioblasts [96–98] and have been the focus of preclinical and initial clinical studies. The potential benefit of these and similar cells [99] has also been linked to their claimed ability to leave the blood vessels after systemic injection. Their potential has been initially tested in mouse [98] and dog [97] models of DMD. In mice, mesoangioblasts derived from mdx animals were corrected using human artificial chromosome-based restoration of the full dystrophin gene [98], while in golden retriever muscular dystrophy (GRMD) dogs, both corrected mesoangioblasts and wild-type cells were injected [97]. Interestingly, autologous mesoangioblasts genetically modified to express dystrophin were less efficient in treating dystrophic dogs than allogeneic ones [97]. Based on these studies, a clinical trial was conducted in five DMD patients who received four intra-arterial injections of HLA-matched mesoangioblasts at 2-month intervals (isolated from the muscles of healthy siblings) [100]. Tacrolimus was administered for immunosuppression, and muscle biopsies analyzed two months after the last injection revealed only low Levels of donor DNA in 4 out of 5 patients. Dystrophin expression was detected in only one case. These results suggest that the efficacy of systemic injection of these cells is quite low and requires substantial improvement. A more detailed review of the mesoangioblast-based approaches has been recently published [101].

The potential of other cell types to enter the muscles after systemic injection was also demonstrated in canine models. Allogeneic muscle stem cells (MuStem cells), distinct from Pax3/Pax7 satellite cells and exhibiting a heterogeneous phenotype (predominantly CD56^+^), were injected systemically (intra-arterially) into immunosuppressed GRMD dogs, resulting in myofiber regeneration, satellite cell replenishment, and improved physical properties of the treated animals [102]. The long-term dystrophin expression was evident, but it was lower than after intramuscular injection. In another study, the same group showed that vascular delivery of allogeneic stem cells in dystrophic dogs requires only short-term immunosuppression, which they ascribed to the low expression of MHC class I antigen [103] as well as the cells’ ability to repress the T-cell proliferation and cytotoxicity [104], a finding observed in human MuStem cells in vitro as well.

Finally, a similar potential in promoting muscle regeneration has been demonstrated for human CD56⁺ MuStem cells transplanted into injured mouse muscles [99]. However, the origin of these cells and their relation to satellite cells is unclear, as they do not express Pax7. If these human myogenic cells represent the same population as canine MuStem cells, which were shown to be effective after intra-arterial delivery [102], they may correspond to mesoangioblast-like cells. Recently, human MuStem cells have been demonstrated to fuse with the nonhuman primate myofibers [105]; however, clinical trials with these cells have not yet been performed.

### Pluripotent stem cell-based therapies

In light of the various characteristics of different and numerous cell types used in experimental therapies for DMD (see table S1 in [99]) and the challenges of translating such studies to humans, it may be reasonable to rely on cell types that can be clearly and unequivocally characterized. The application of pluripotent stem cells (PSCs), either embryonic stem cells (ESCs) or induced pluripotent stem cells (iPSCs), represents an attractive option, as they can be efficiently differentiated into muscle cells through genetic overexpression of Pax3/Pax7 [106–108] or small molecule-driven protocols [40, 109, 110] (Table 2). However, this will require overcoming the technical and medical issues associated with these cells.

**Table 2:** Examples of pluripotent cell-based approaches to correct dystrophin deficiency in animal models of Duchenne muscular dystrophy

Pluripotent cell	Mode of differentiation	Animal model of DMD	Number and phenotype of cells applied for therapy	Effect after transplantation	References
hESC	Embryoid body differentiation & DOX-induced Pax3 expression	*mdx* mice; tacrolimus for immunosuppression	1.5 × 10^6^ cells, sorted for PDGF-αR^+^/Flk-1^−^; intramuscular (TA) and systemic (i.v. and i.a) injection	Engraftment, also after systemic injection, although to a lesser extent (note: non-sorted Pax3-induced cells formed teratomas)	[106]
hESC	Culture in myogenic medium followed by transduction with adenoviral MyoD vector	Rag/*mdx* mice	5 × 10^5^ MyoD^+^ cells into the muscles	Myofibers expressed dystrophin	[188]
hESC hiPSC	DOX-induced Pax7 expression	NSG-*mdx*^4Cv^ mice	5 × 10^5^ Pax7^+^ myogenic progenitors	Persistent engraftment for 11 months after transplantations and repopulation of the satellite cell compartment	[189]
mESC	Serum-free protocol (GSK3β inhibitor: CHIR99021, BMP inhibitor: LDN193189, IGF-1, HGF, FGF-2)	Rag1^−/−^Dmd^*mdx*−5Cv^ mice	Pax7-GFP cells (artificial chromosome); 1 × 10^5^ into the TA muscle	Foci of dystrophin^+^ and MyHC^+^fibers visible one month after injection; Pax7^+^ satellite-like cells detected under the basal lamina of GFP^+^ fibers	[40]
hiPSC	Transgene-free or MyoD expression and correction of DMD mutation (CRISPR/Cas9 knockout of exon 45–55)	NSG-*mdx* mice	1 × 10^6^ NCAM ^+^ HNK1^−^ cells injected intramuscularly	Few donor-derived dystrophin-positive myoblasts demonstrated engraftment; efficacy improved in the subsequent study of this group [190]	[191]
hiPSC	CHIR99021-DAPT culture: GSK3β inhibition followed by Notch signalling inhibition; Genetic restoration of dystrophin by artificial chromosome	NSG-*mdx*^4Cv^ mice NOD-SCID-IL2rγ^null^-*mdx*^4Cv^ mice (immunodeficient mice lacking dystrophin)	1–3 × 10^6^ CD57^+^ (NCAM^+^) HNK1^−^cells	Contribution to myofibers	[192]
hESC hiPSC	DOX-induced Pax7 expression (as in [189])	NSG-*mdx*^4Cv^ mice	5 × 10^5^ CD54, α9β1 integrin, and syndecan 2-purified Pax7^+^ progenitors	Efficient engraftment	[193]
hESC	Serum-free protocol	NSG-*mdx*^4Cv^ mice	3 × 10^5^ CD10^+^CD24^−^ myogenic progenitors into CTX-injured muscles	12% donor-derived satellite cell engraftment	[117]
hPSC	Serum-free protocols (two modes compared)	NSG-*mdx* mice	1 × 10^6^ ERRB3^+^ or NGFR^+^ myogenic progenitors (dystrophin expression restored by CRISPR/Cas9 correction)	Enrichment for ERBB3 and NGFR enriched for Pax7^+^ fetal and PSC-derived cells and improved engraftment of PSC; PCS-derived muscle progenitors less effective than CD45^−^CD31^−^NCAM^+^ fetal satellite cells (PSC-muscle progenitors did not fuse with the host fibers)	[190]
hiPSC	Transgene-free differentiation	NSG-*mdx* mice	1–2 × 10^6^ MYF5^+^ cells; muscles were not CTX-treated	Engraftment; Functional recovery of DMD muscle at 6 weeks after injection	[113]
hiPSC	Transgene-free differentiation	NOG-*mdx* mice	1 × 10^5^ NGFR^+^ cells	Better engraftment than negative cells	[118]
hiPSC	Serum-free protocol	NSG-*mdx*^4Cv^ mice	1 × 10^6^ Pax7:GFP cells	Long-term regenerative potential and maturation of the cells upon in vivo engraftment; In the non-*mdx* model, the regenerative potential in the secondary recipient demonstrated	[110]
hESC hiPSC	DOX-induced Pax7 expression, integration into safe harbor locus	NSG-*mdx*^4Cv^ mice	1 × 10^6^ CD54 and syndecan 2 -purified Pax7^+^ progenitors	Efficient engraftment (2 months)	[194]
hiPSC	DOX-induced Pax7 expression (as in [194])	NSG-*mdx* mice	1.65 × 10^6^ iPax7^+^ cells injected into the TA and quadriceps	Long-term (2 months) engraftment; transcriptional maturation	[120]
hiPSC	Pax7 lentiviral overexpression; correction of mutation by CRISPR/Cas9 knock-in to connect exon 44 to exon 58	NSG-*mdx*^4Cv^ mice	1 × 10^6^ Pax7^+^ myogenic progenitors transplanted into irradiated TA muscle	Donor-derived myofibers produced	[119]
Teratoma-derived myogenic progenitors	Formation of teratoma in the TA muscle for 4 weeks; isolation of myogenic progenitors	NSG-*mdx*^4Cv^ mice	2–4 × 10^5^ α7^+^ VCAM^+^ myogenic progenitors isolated from teratomas (for teratoma formation, 2.5 × 10^5^ ESCs or 1 × 10^6^ were injected into the TA muscle)	Very efficient engraftment (~ 80%) of the regenerating TA muscle; Matured after transplantation into quiescent muscle stem cells	[115]
Teratoma-derived myogenic progenitors	In vitro expanded progenitors obtained as in [115]; nine orders of magnitude expansion for 37 days	NSG-*mdx*^4Cv^ mice	4 × 10^5^ α7^+^ VCAM^+^ myogenic progenitors isolated from teratomas (for teratoma formation, 2.5 × 10^5^ ESCs were injected into the TA muscle)	Efficient engraftment (40%); functional improvement of the transplanted muscles	[116]

*Abbreviations: BMP *bone morphogenetic protein, *CTX *cardiotoxin,* DOX *doxycycline, *FGF-2 *fibroblast growth factor-2, *GSK3β *glycogen synthase kinase-3 beta, *hESC *human embryonic stem cells,* HGF *hepatocyte growth factor, *iPAX7 *doxycycline (dox) inducible expression of PAX7,* hiPSC *human induced pluripotent stem cells, *i.a. *intra-arterial, *IGF-1* insulin-like growth factor-1, *i.v. *intravenous, *mESC *murine embryonic stem cells, *NGFR *nerve growth factor receptor, *PDGF-αR*platelet-derived growth factor α receptor,* TA* tibialis anterior, *VCAM-1 *vascular cell adhesion protein 1, *α7 *α7-integrin

In their initial study, Perlingeiro’s group demonstrated that ESC-derived platelet-derived growth factor alpha receptor-positive and Flk-1-negative cells (PDGF-αR^+^/Flk-1^−^) (hence not endothelial type) possess muscle regenerative potential [106]. Such cells, when injected intramuscularly or systemically (intravenously or intra-arterially) into dystrophic mice, engrafted into the muscles and improved their contractile potential. Subsequently, in 2013, the same group conducted a feasibility study demonstrating the muscle-regenerating potential of iPSC-derived cells in a murine DMD model. In that work, Filareto et al. [111] generated iPSCs from fibroblasts of mice lacking both dystrophin and utrophin, and then they restored the microutrophin (µUtrn) expression, a short version of utrophin, using the Sleeping Beauty transposon system. Modified iPSCs were differentiated into PDGF-αR^+^ skeletal muscle progenitors, which, when transplanted into dystrophic mice, engrafted into the muscle, generating a large number of utrophin-positive myofibers and improving contractile strength. The authors also reported the presence of utrophin-positive fibers in the skeletal muscles and occasionally in the diaphragm after systemic (intravenous) injection of the PSC-derived muscle progenitors, but it was undetected in the hearts of the injected mice [111]. This might indicate that such progenitors can leave blood vessels and enter the muscle. However, the efficacy of that has not been evaluated. Importantly, before cell administration, the mice were also injected with the cardiotoxin to induce muscle injury, a condition that may have facilitated engraftment, as shown previously [102].

The application of PSC-derived muscle progenitors has also been demonstrated in several other studies [40, 109, 112–117], (see also Table 2 for additional references). However, the methods of differentiation show various efficacies, and some of the proposed modalities rely on gene transfer-based overexpression of myogenic factors. Similar to the use of primary satellite cells (described above), the long-term culture of muscle progenitors may limit their therapeutic efficacy. Therefore, future application of PSC-derived myogenic progenitors may require the selection of the proper progenitor cell population to ensure an efficient method for propagating a sufficient number of cells for the therapy. This may be particularly crucial for human progenitor cells, as it has been demonstrated that mSCs lose engraftment capacity after long-term culture. A recent study suggested that CDH13 (T-cadherin or H-cadherin) and fibroblast growth factor receptor 4 (FGFR4) could serve as markers for human myogenic cell purification, with these cells contributing to muscle regeneration in *mdx* mice [118]. Additionally, Zhao et al. [113] found that human iPSC-derived Myf5^+^ cells, when injected intramuscularly into immunodeficient *mdx* mice, recovered dystrophin expression and improved muscular function.

A recent proof-of-principle study by Dhoke et al. [119] demonstrated the therapeutic potential of patient-specific iPSCs. The authors generated iPSCs from two individuals carrying mutations in exons 45 and 51 of the *DMD* gene and successfully corrected these mutations using the CRISPR/Cas9 system. The gene-corrected iPSCs were then differentiated into myogenic progenitors and transplanted into the muscles of immunodeficient dystrophic NSG-*mdx*^4cv^ mice, resulting in successful engraftment and dystrophin expression [119]. However, functional improvements in muscle performance were not reported. Notably, iPSC-derived Pax7⁺ progenitors have been shown to undergo transcriptional maturation following transplantation into both dystrophic and non-dystrophic muscle environments [120]. Importantly, the human muscle progenitor Pax7-expressing cells, generated by chemical-driven differentiation, efficiently engrafted into the damaged muscles of NSG-*mdx*^4cv^ mice [110], improving the exercise capacity of the mice in the treadmill assay and contributing to the quiescent satellite cell population. Such in vivo matured satellite cells demonstrated the ability to regenerate muscle upon reinjury and to repopulate the muscle niche in secondary recipient mice [110].

Importantly, this study [110] provides proof of principle for the therapeutic potential of iPSC-derived mSCs, which can mature efficiently upon in vivo engraftment, in contrast to in vitro conditions. However, challenges remain in generating a large number of these cells and achieving effective long-term engraftment, particularly after systemic delivery, which remains inefficient for this type of muscle progenitor. Recently, the FDA approved the investigational use of iPSC-derived myogenic progenitors in non-ambulant DMD patients [121], using cells generated through a newly published protocol [108].

Interestingly, Kyba’s team [115] proposed an alternative strategy for generating large numbers of progenitor cells. The authors injected PSCs into the mouse muscle, which resulted in the formation of teratomas. Then, the α7integrin^+^VCAM-1^+^ myogenic progenitors have been FACS-purified from such teratomas, and following injection, these cells demonstrated high engraftment in injured muscle, ameliorating muscular dystrophy [115]. Importantly, such progenitors contributed to the generation of the quiescent Pax7^+^ satellite cells, which responded to subsequent injuries by regenerating muscles. In their follow-up study, the authors successfully propagated these progenitors in vitro, thereby expanding the therapeutic potential of the proposed method while preserving the regenerative capacity of the Pax7^+^-teratoma-derived cells [116].

Recently, the same approach has been tested for the human teratoma-derived progenitors [122], achieving successful in vitro expansion of the CD82^+^ERBB3^+^NGFR^+^ skeletal myogenic progenitor population. Of note, the expression of CD82 (tetraspanin) could serve as a prospective marker for isolating human satellite cells [123]. The results proved the feasibility of testing iPSC-derived muscle progenitors in DMD patients; however, clinical studies have not yet been initiated due to technical constraints and safety concerns. Progress could be expected if the efficient differentiation of progenitors with stable regenerative properties is achieved with minimal genetic manipulation, ideally with transgene-free protocols. In recent years, several such protocols have been proposed and tested [124, 125]. However, the potential of these cells remains to be determined, particularly regarding their stability and survival [126]. A crucial issue will be achieving therapeutic success after systemic delivery, as intramuscular injections can provide only localized improvement, if any. It remains to be established whether PSC-derived muscle progenitors can effectively exit the bloodstream and engraft into the muscle tissue. As noted, such properties are absent for human myoblasts but have been suggested for mesoangioblasts ([97]; for a review, see [101, 127]).

The studies showing the successful engraftment of PSC-derived muscle progenitors were preceded and likely facilitated by muscle irradiation and cardiotoxin injury [122]. Such an approach would be unacceptable in humans, and it is uncertain to what extent the endogenous injury caused by the lack of dystrophin would facilitate cell homing in DMD patients. PSCs may also enable the investigation of the systemic delivery of the myogenic precursors. Through appropriate selection, treatment, or genetic modification, it may be possible to identify cells expressing specific markers that promote or improve their passage through blood vessels into the muscle. In line with such a possibility, Choi et al. [128] recently showed that treatment of CD56^+^ myogenic progenitors with DLL4 and PDGF-BB improves the migration of hiPSC-derived myogenic progenitors.

### Other cell types

In addition to satellite cells, vessel-associated mesangioblasts, and PSC-derived myoblasts, several other cell types have been proposed as potential therapies for DMD [127]. These applications are often based on claims that these cells can differentiate into muscle. However, it is crucial to critically evaluate the scientific rationale behind the studies suggesting the therapeutic efficacy of so-called mesenchymal stem/stromal cells (MSCs) and chimeric cells to determine their validity.

#### Mesenchymal “stem” cells

A widespread misconception has arisen from mesenchymal stromal cell transplantations, commonly but inaccurately referred to as mesenchymal stem cells (hence both abbreviated as MSCs). These cells have been asserted to possess multilineage differentiation potential, enabling them to repair muscle, heart, and other tissues across various diseases. This claim is based on the belief that MSCs undergo differentiation into numerous cell types, including skeletal muscle cells or cardiomyocytes, thereby offering therapeutic avenues for muscular diseases. However, while bone marrow-derived MSCs are capable of differentiating into adipocytes, chondrocytes, and osteoblasts, this does not extend automatically to differentiation into other cell types. Various stromal cells from different organs demonstrate tissue-specific differentiation capacity (for references see: [129, 130]). Their claimed wide differentiation capabilities have been questioned and often attributed to experimental artifacts [129–131]. Some studies have even claimed that allogeneic, adipose tissue-derived MSCs were therapeutically effective in immunocompetent *mdx* mice [132] and that these cells could persist for several months in non-immunosuppressed GRMD dogs after local or systemic administration [133]. However, these findings have not been reproduced, and their translation to human patients remains highly questionable. The variability in disease progression among DMD dogs, along with concerns about the quality and controls of these studies, raises doubts about the effectiveness of this approach. It has to be stressed that the perpetuated claims on muscle-differentiation properties of various MSCs have been disproven in a landmark study by Paulo Bianco’s group. This research demonstrated that CD146^+^ stromal cells isolated from human muscle could differentiate into myoblasts in a rigorous in vivo assay, while this was not the case for bone marrow, periosteum, or cord blood-derived CD146^+^ cells [134]. This study highlighted that tissue-specific stem/progenitor cells (generally referred to as MSCs) are restricted to differentiating into cell types native to their tissue of origin.

Analysis of outcomes following the use of other “popular” cells, such as umbilical cord Wharton’s jelly-derived cells, also named MSCs, indicates their ineffectiveness in treating conditions like DMD, as discussed in a recent review [135]. Surprisingly, studies on this approach are continued, and it is being promoted as a potential pathway for conditional drug approval.

In sum, the so-called MSCs isolated from several tissues (bone marrow, adipose, Wharton’s jelly of the umbilical cord, teeth, etc.) represent different stromal-cell types with various origins. Their stem cell properties are at best disputable, as such claims are based on assays prone to artifacts (for references, see [129, 134, 136–138]). Unfortunately, these cells are commonly used and even offered commercially as an unregulated and unproven treatment for numerous conditions (for references, see [136–138]).

#### Chimeric cells

An even more unjustified example of a questionable strategy is the use of so-called chimeric cells. This approach will be examined in greater detail, particularly because it recently received orphan drug designation from the FDA despite unclear justification and well-founded criticism [139].

The concept of using chimeric cells is presented in several papers from one research group, and a clinical trial has also been initiated [140]. According to this strategy, the patient’s myoblasts (lacking dystrophin) are combined (“fused”) in the laboratory with the father’s dystrophin-expressing myoblasts, forming so-called dystrophin-expressing chimeric (DEC) cells, which are then administered intraosseously into the patient’s bones. Proponents of this approach claim that this unusual, uncommon, and invasive route of cell delivery is designed to induce immunological tolerance to the father’s antigens, potentially including dystrophin. However, a detailed review of the published experimental work in animal models, which, according to the authors, proved the feasibility of the approach, fails to substantiate any of their proposed hypotheses.

The concept of using chimeric cells is presented in several papers from one research group, and a clinical trial has also been initiated [140]. According to this strategy, the patient’s myoblasts (lacking dystrophin) are combined (“fused”) in the laboratory with the father’s dystrophin-expressing myoblasts, forming so-called dystrophin-expressing chimeric (DEC) cells, which are then administered intraosseously into the patient’s bones. Proponents of this approach claim that this unusual, uncommon, and invasive route of cell delivery is designed to induce immunological tolerance to the father’s antigens, potentially including dystrophin. However, a detailed review of the published experimental work in animal models, which, according to the authors, proved the feasibility of the approach, fails to substantiate any of their proposed hypotheses.

First, the initial studies were conducted in systems that inherently prevent cell rejection. For example, mdx mice received myoblasts from genetically identical healthy strains, or immunodeficient SCID/mdx mice were transplanted with human myoblasts, which are not rejected in these hosts. Second, and more importantly, these studies did not show the presence of injected cells in the circulation following intrabone administration, an essential step if subsequent muscle engraftment were to occur [140–142]. Third, even if myoblasts could enter the circulation from the bone marrow (which has not been demonstrated), numerous studies have evidenced that they do not migrate from the vasculature into muscle tissue (as discussed above).

The presence of such chimeric myoblasts in the muscles or heart, alongside the reported functional improvements, raises serious concerns. Recently, the administration of chimeric cells to three patients - two six-year-old walking boys and one 15-year-old boy using a wheelchair was reported [140]. The authors claimed that six months after the intraosseous administration of chimeric myoblasts, some symptoms showed signs of improvement. However, the open, non-randomized nature of the studies, which does not account for any accidental changes that may occur in patients, does not allow these results to be considered conclusive evidence of a therapeutic effect.

Moreover, the unproven validity of the hypothesis of administering myoblasts intraosseously, i.e., injecting cells into a site where they do not naturally reside, raises serious methodological and safety concerns. The concept of such invasive treatments, which may create the illusion of potential effectiveness, also brings up ethical issues. Both experimental and clinical applications of this approach have recently been scrutinized by experts in the field. A comprehensive analysis, published on the website of the DMD patients organization, World Duchenne, warns patients about this scientifically, medically, and ethically questionable strategy (see recent comment by Aartsma-Rus [139]).

Unfortunately, some researchers continue to pursue and promote the discredited effects of these cells. This raises ethical concerns about such claims, especially given their lack of scientific validity, methodological rigor, and demonstrable benefit to patients. Generating hype and offering false hope in the absence of a credible therapeutic effect can ultimately be more harmful than providing no intervention at all.

## Cell therapies for the treatment of DMD-related cardiomyopathy

The heart does not contain cardiac stem cells. Although various cell-based therapies have been proposed and even clinically tested in patients with heart failure, in light of the negative results (described above), the rationale and justification currently rely on the application of PSCs, which are differentiated into cardiomyocytes before being injected into the heart. However, some other modalities have been suggested in the pursuit of improving heart function. As in skeletal muscles, it has been proposed that the small vessels of the juvenile mouse ventricle contain mesoangioblasts [143]. Aorta-derived mesoangioblasts, characterized by the expression of Nkx2.4, cardiac troponin I, tropomyosin, and α-actin, have been injected into the hearts of young mdx mice [144]. The authors reported that these cells appeared to differentiate into dystrophin-expressing cardiomyocytes and could enhance blood vessel formation, as evidenced by the higher expression of CD31. However, the approach was inefficient in the fibrotic hearts of aged mdx mice, resulting in no functional improvement [144]. Nevertheless, the properties of mesoangioblasts and their contribution to heart repair raise concerns, particularly in light of other studies indicating the lack of progenitor cells in the adult heart.

Cardiosphere cells, isolated from the heart, are considered a type of stromal progenitor cell with claimed immunomodulatory, antifibrotic, and regenerative actions in DMD (for references, see [145]). The clinical studies were based on preclinical work, where mdx mice, systemically injected with these cells, demonstrated improvements in cardiac and skeletal muscle structure and function [145]. These beneficial effects were ascribed to the exosomes [146], which, when secreted from cardiospheres, are claimed to reprogram macrophages and fibroblasts and to affect endogenous cardiac progenitors. A recent study demonstrated that both mouse cardiosphere-derived cells (mCDCs) and extracellular vesicles (EVs) secreted by human cardiosphere-derived cells (hCDC-EVs) exerted a long-term effect on DMD pathology in mdx mice [147]. When young (8-week-old) mice were injected with mCDCs once monthly over a one-year study period, muscle function and structure were preserved, protecting against functional decline even at 12 months of age. In contrast, the efficacy of hCDC-EVs declined after six months of treatment, likely due to the activation of an immune response that resulted in their immunological clearance and a corresponding reduction in therapeutic benefit [147].

These findings prompted the initiation of clinical trials. In the HOPE-Duchenne trial (NCT02485938, ClinicalTrials.gov), 25 non-ambulant patients with a mean age of 17.8 years were randomized to receive an intracoronary infusion of CDCs (CAP-1002) (13 patients) or serve as controls (12 patients) [148]. MRI performed 12 months after treatment revealed significant scar reduction, improvements in inferior wall systolic thickening, and enhanced mid-distal upper limb performance [148]. In the HOPE-2 multicenter Phase 2 trial (NCT03406780, ClinicalTrials.gov), mostly non-ambulant patients were randomized. Eight patients (seven non-ambulant and one in the late ambulant stage) were administered intravenous injection of allogeneic CDCs, derived from donor hearts. Each patient received 1.5 × 108 cells, once every three months, for a total of four infusions, with a 12-month follow-up.

A total of twelve patients were randomized to receive a placebo; however, six of them were excluded from the final analysis due to screening failures. The study concluded that patients treated with CAP-1002 showed stabilization of upper limb function and improvements in cardiac function and structure [149]. However, the study was subsequently discontinued by the sponsoring company, Capricor Therapeutics, with the claims of financial issues. Despite reporting positive outcomes, the approach was associated with serious adverse events, such as strong hypersensitivity reactions in three patients within the treatment group. This led to the protocol modification, requiring all patients to receive glucocorticoids, as well as H1 and H2 blockers [150]. The authors stated, based on previous preclinical studies, that CDCs do not express MHC class II surface antigens or the costimulatory molecules CD80 and CD86. However, the occurrence of hypersensitivity reactions raises concerns about the safety of this approach. Furthermore, the mechanisms underlying the observed cardiac benefits remain unclear, aside from the acclaimed paracrine effect, which has not, however, been conclusively proven. The proposed exosome-mediated profits are still speculative and poorly understood, leaving their true mechanisms of action uncertain. Despite these challenges, recent reports from Capricor Therapeutics suggest that a follow-up study indicates improvements in cardiac function in cardiosphere-treated patients. Based on these findings, the company planned to seek FDA approval [151], however, in July, the FDA rejected the application and did not grant registration [152]. A subsequent trial is currently ongoing (HOPE-3, NCT05126758; ClinicalTrials.org), evaluating the safety and efficacy of allogeneic hCDCs, now referred to as deramiocel, in patients with advanced-stage DMD.

It can be presumed that the PSC-based approaches developed for treating heart failure, which have also been recently tested in humans [153], could potentially be applied in DMD patients. PSCs are the only cells that can be effectively differentiated into cardiomyocytes. The transplantation of either ESC- or iPSC-derived cardiomyocytes has been tested in various animal models of heart failure, including studies in non-human primates (for reviews, see [154–157]). However, applying cardiac cell therapy to DMD patients may be particularly challenging in the advanced stages of the disease. A critical barrier is the overall health status of DMD patients, who are generally considered ineligible for heart transplantations. The same challenges could restrict the application of cell therapy in DMD hearts.

An additional issue limiting the efficacy of cell therapies in DMD is the persistence of the damaged cells in the muscles and heart, where the therapeutic cells are intended to be injected. It is important to note, that to date, the only effective cell therapies require ablation of diseased cells, as is the case in hematopoietic stem cell (HSC) transplantation for blood diseases, or removal of damaged cells, as in burns or treatment of damaged corneal epithelium to provide a favorable environment for the therapeutic cells to take hold and regenerate tissue effectively (for a review, see [138]). The application of cell therapies to severely damaged skeletal and cardiac muscles exposes transplanted cells to unfavorable environments, limiting their survival and therapeutic efficacy. Therefore, combining cell-based approaches with anti-inflammatory and anti-fibrotic treatments emerges as a rational strategy.

## Cell therapies for other forms of muscular therapies

DMD represents the most common and remains the most studied form of muscular dystrophy, yet other genetically inherited muscle disorders, such as Becker muscular dystrophy (BMD), limb-girdle muscular dystrophies (LGMD), facioscapulohumeral dystrophy (FSHD), oculopharyngeal muscular dystrophy (OPMD) and congenital forms like LAMA2-related muscular dystrophy, also present significant unmet medical needs. While these diseases share core features like progressive muscle weakness, yet differ markedly in their genetic origins (with the exception of BMD and DMD, which are both caused by mutations in the *DMD* gene), age of onset, severity, and progression. This diversity presents a considerable challenge in developing universally applicable therapeutic strategies. Among emerging treatments, cell therapy holds promise across multiple dystrophy types. However, most efforts remain at the preclinical stage.

In the experimental approaches (with exemplary strategies for LGMD and FSHD summarized in Table 3), various cell types were used to replace the damaged muscles. Each disease represents specific and different challenges from DMD (except for BMD, which is, in fact, usually a milder form of DMD), and it is beyond the scope of this paper to discuss them in detail. Recent reviews highlighted emerging therapies in various muscular dystrophies have been published [158, 159]. Readers seeking more in-depth information are encouraged to consult these review papers and the original studies referenced in Table 3.

**Table 3:** Examples of cell replacement therapies for muscular dystrophies other than DMD in experimental animal models

Dystrophy type	Subtype/ abbreviation*	Mutation (gene)	Cell type used for therapy	Model	Therapeutic effect	References
Limb girdle muscular dystrophy	LGMDR9 FKRP-related (LGMD2I)	*FKRP*	FKRP L276I^KI^-mouse derived satellite cells; lentiviral expression of WT human *FKRP* gene	FKRP L276I^KI^mice	Recovery of α-DG glycosylation; improvement of muscle strength	[195]
	LGMDR9 FKRP-related (LGMD2I)	*FKRP*	mESC- and hiPSC-derived myogenic progenitors	FKRP P448L-NSG mice	Enhanced specific force in transplanted muscle	[196]
	LGMDR9 FKRP-related (LGMD2I)	*FKRP*	CRISPR-Cas9 corrected LGMDR9 iPSC-derived progenitors	FKRP P448L-NSG mice	Myofiber and satellite cell engraftment, restoration of α-DG functional glycosylation	[197]
	LGMDR3 α-sarcoglycan-related (LGMD2D)	*SGCA*	Patient-iPSC-derived-mesoangioblasts; lentiviral restoration of α-sarcoglycan	*Sgca*-null/scid/beige mice (dystrophic, immune-deficient triple mutant)	Functional amelioration of dystrophic phenotype, restoration of depleted progenitors	[198]
	LGMDR1 calpain3-related (LGMD2A)	*CAPN3*	CRISPR-Cas9 corrected LGMDR1 iPSC-derived progenitors	Immunodeficient *CAPN3-*KO mice	Muscle engraftment, rescue of *CAPN3* mRNA	[160]
	LGMDR1 calpain3-related (LGMD2A)	*CAPN3*	CRISPR/Cas9 corrected LGMDR1 iPSC-derived progenitors	Immunodeficient *CAPN3-*KO mice	*CAPN3* expression evident after cell transplantation	[161]
	LGMDR21 POGLUT-1-related (LGMD2U)	*POGLUT1*	CRISPR-Cas9 corrected LGMDR21 iPSC-derived progenitors	NSG-immunodeficient WT mice; cardiotoxin injury of muscle	Rescue of impaired engraftment exhibited by mutated cells	[199]
	LGMDR2 dysferlin-related (LGMD2B)	*DYSF*	CRISPR-edited murine primary muscle stem cells	hEx44mut mice (humanized mouse model with *Dysf* exon 44 with the founder frameshift mutation; autologous therapy)	Regeneration of muscles; repopulation of the muscle stem cell niche	[200]
	LGMD R2 dysferlin-related (LGMD2B)	*DYSF*	Healthy human and mouse myoblasts	SCID and SJL mice (dysferlinopathy model)	Dysferlin-positive fibers detected in muscle sections; the therapeutic effect limited by poor cell migration	[201]
Facioscapulohumeral muscular dystrophy	FSHD	*DUX4* overexpression due to defective epigenetic repression	Mouse ESC-derived muscle progenitors with an induced low level of DUX4	iDUX4pA-HSA mice (doxycycline-induced *DUX4* expression)	Reduced fibrosis and improved contractile force with myogenic progenitors expressing a low level of DUX4	[163]
	FSHD	*DUX4* overexpression due to defective epigenetic repression	Mouse ESC-derived myogenic progenitors	FRG1 transgenic mice	Functional improvement in muscles of treated male mice (more severely affected than females)	[202]

*Abbreviations:CAPN3 *calpain 3,* DYSF *dysferlin,FRG1 FSHD Region Gene 1*, FRKP *fukutin-related protein, *hiPSC* human induced pluripotent stem cells,* mESC *murine embryonic stem cells,* POGLUT1 *O-glucosyltransferase 1,* SGCA * α-sarcoglycan

New and old (in brackets) LGMD subtype nomenclature was given according to Straub et al. [203], and subsequent reports

Based on the experimental studies, a few clinical trials have been recently proposed. The bASKet trial (NCT05588401, ClinicalTrials.gov) is a Phase I/IIa study evaluating the safety of CRISPR/Cas9-corrected autologous satellite cells (GenPHSats) over a 12-month monitoring period in six LGMD patients. The idea is based on the initial works by Selvaraj et al. [160] and Müthel et al. [161], demonstrating the utility of the CRISPR/Cas9 approach to correct mutations in the calpain-3 (*CAPN3*), the leading cause of the LGMDR1 **(**Table 3**)**. A similar strategy, VTA-100 by Vita Therapeutics, targeted *CAPN3* mutations was cancelled in 2024 due to financial constraints, despite promising preclinical results. The company has since shifted focus to VTA-200, an allogeneic, hypoimmunogenic satellite cell therapy, aiming for broader, off-the-shelf application [162]. It also aims to develop universal iPSC-based therapy for FSHD, targeting double homeobox 4 (DUX4)-driven toxicity. Supporting this strategy, Azzag et al. [163] **(**Table 3**)** demonstrated that DUX4-silenced iPSC-derived myogenic cells reduced fibrosis and improved disease features in an FSHD mouse model.

Encouraging clinical results have been reported for OPMD, owing to the localized nature of the disease. In this form of dystrophy, caused by mutations in the *PABPN1* gene, the pathology primarily affects a limited set of muscles, including the levator palpebrae, pharyngeal muscles, and proximal limb muscles. This restricted muscle involvement presents a unique therapeutic opportunity: autologous genetically normal myoblasts can be harvested from healthy and functionally intact muscles and locally transplanted into diseased areas without requiring genetic modification or immunosuppressive therapy. This approach was validated by Perié et al. [164], who conducted a phase I/IIa clinical study (NCT00773227, ClinicalTrials.gov) involving 12 OPMD patients. In this study, autologous myoblasts, isolated from unaffected quadriceps or sternocleidomastoid muscles, were injected into the pharyngeal muscles. Over a 2-year follow-up, no adverse effects were reported, while an improvement in the quality of life score was found for all 12 patients, and a cell dose-dependent improvement in swallowing function was observed for 10 patients. However, no recent data are available.

## Future directions

Cellular therapies are regarded as potential interventions to address the pathological mechanisms of DMD. However, unlike the well-established application of cell therapy in hematological disorders, their systemic efficacy in muscle diseases remains limited due to the widespread and extensive nature of the affected tissues. Notably, the volume of the tissue requiring treatment is enormous, presenting a significant logistical and biological challenge. Moreover, the success of HSC therapy is partly attributed to the removal (ablation) of diseased cells before transplantation, a step that is not feasible in DMD. In contrast, cell therapies for DMD are introduced into a hostile microenvironment characterized by chronic inflammation, fibrosis, and ongoing muscle fiber necrosis in both skeletal and cardiac tissues. These conditions severely constrain the therapeutic potential of transplanted cells, making them markedly less effective than HSC transplantation following myeloablation.

A promising future strategy to target cardiac fibrosis may involve chimeric antigen receptor-T cell (CAR-T) technology, which has demonstrated preclinical efficacy in models of hypertensive cardiomyopathy and potential utility for cardiac repair [165–167]. CAR-T targeting fibroblast activation protein (FAP), currently considered for treating solid tumors [168], may represent a future direction in DMD therapy. To our knowledge, such a scenario has not been extensively (or almost has not been) explored in the context of DMD-related cardiomyopathy, except for the initial work by Marigny et al. [169] (available only in abstract form), who reported the beneficial effects of FAP-CAR-T cell therapy in lymphodepleted D2.*mdx* mice. The potential benefits of combining cell therapy with anti-inflammatory and anti-fibrotic approaches in other mouse models and DMD patients remain to be experimentally validated.

## Data Availability

No datasets were generated or analyzed for this manuscript.

## References

[1] Bladen CL, Salgado D, Monges S et al (2015) The TREAT-NMD DMD global database: analysis of more than 7,000 Duchenne muscular dystrophy mutations. Hum Mutat 36:395–402. 10.1002/humu.22758 25604253 10.1002/humu.22758 PMC4405042

[2] Le S, Yu M, Hovan L et al (2018) Dystrophin as a molecular shock absorber. ACS Nano 12:12140–12148. 10.1021/acsnano.8b05721 30457830 10.1021/acsnano.8b05721 PMC6632074

[3] Ramaswamy KS, Palmer ML, van der Meulen JH et al (2011) Lateral transmission of force is impaired in skeletal muscles of dystrophic mice and very old rats. J Physiol 589:1195–1208. 10.1113/jphysiol.2010.201921 21224224 10.1113/jphysiol.2010.201921 PMC3060596

[4] Allen DG, Whitehead NP, Froehner SC (2016) Absence of dystrophin disrupts skeletal muscle signaling: roles of Ca2+, reactive oxygen species, and nitric oxide in the development of muscular dystrophy. Physiol Rev 96:253–305. 10.1152/physrev.00007.2015 26676145 10.1152/physrev.00007.2015 PMC4698395

[5] Constantin B (2014) Dystrophin complex functions as a scaffold for signalling proteins. Biochimica et Biophysica Acta (BBA) 1838:635–642. 10.1016/j.bbamem.2013.08.023 24021238 10.1016/j.bbamem.2013.08.023

[6] Loufrani L, Matrougui K, Gorny D et al (2001) Flow (shear stress)-induced endothelium-dependent dilation is altered in mice lacking the gene encoding for dystrophin. Circulation 103:864–870. 10.1161/01.cir.103.6.864 11171796 10.1161/01.cir.103.6.864 PMC2233878

[7] Palladino M, Gatto I, Neri V et al (2013) Angiogenic impairment of the vascular endothelium: a novel mechanism and potential therapeutic target in muscular dystrophy. Arterioscler Thromb Vasc Biol 33:2867–2876. 10.1161/ATVBAHA.112.301172 24072696 10.1161/ATVBAHA.112.301172

[8] Loufrani L, Dubroca C, You D et al (2004) Absence of dystrophin in mice reduces NO-dependent vascular function and vascular density: total recovery after a treatment with the aminoglycoside gentamicin. Arterioscler Thromb Vasc Biol 24:671–676. 10.1161/01.ATV.0000118683.99628.42 14751810 10.1161/01.ATV.0000118683.99628.42 PMC2233851

[9] Hugnot JP, Gilgenkrantz H, Chafey P et al (1993) Expression of the dystrophin gene in cultured fibroblasts. Biochem Biophys Res Commun 192:69–74. 10.1006/bbrc.1993.1382 8476435 10.1006/bbrc.1993.1382

[10] Wang Y, Marino-Enriquez A, Bennett RR et al (2014) Dystrophin is a tumor suppressor in human cancers with myogenic programs. Nat Genet 46:601. 10.1038/ng.2974 24793134 10.1038/ng.2974 PMC4225780

[11] Łoboda A, Dulak J (2020) Muscle and cardiac therapeutic strategies for Duchenne muscular dystrophy: past, present, and future. Pharmacol Rep 72:1227–1263. 10.1007/s43440-020-00134-x 32691346 10.1007/s43440-020-00134-x PMC7550322

[12] Bushby K, Finkel R, Birnkrant DJ et al (2010) Diagnosis and management of Duchenne muscular dystrophy, part 1: diagnosis, and pharmacological and psychosocial management. Lancet Neurol 9:77–93. 10.1016/S1474-4422(09)70271-6 19945913 10.1016/S1474-4422(09)70271-6

[13] Lofaso F, Orlikowski D, Raphael J-C (2006) Ventilatory assistance in patients with Duchenne muscular dystrophy. Eur Respir J 28:468–469. 10.1183/09031936.06.00059906 16946091 10.1183/09031936.06.00059906

[14] McDonald DGM, Kinali M, Gallagher AC et al (2002) Fracture prevalence in Duchenne muscular dystrophy. Dev Med Child Neurol 44:695–698. 10.1017/s0012162201002778 12418795 10.1017/s0012162201002778

[15] Politano L, Nigro V, Nigro G et al (1996) Development of cardiomyopathy in female carriers of Duchenne and Becker muscular dystrophies. JAMA 275:1335–1338 8614119

[16] Song T-J, Lee K-A, Kang S-W et al (2011) Three cases of manifesting female carriers in patients with Duchenne muscular dystrophy. Yonsei Med J 52:192–195. 10.3349/ymj.2011.52.1.192 21155054 10.3349/ymj.2011.52.1.192 PMC3017697

[17] Ishizaki M, Kobayashi M, Adachi K et al (2018) Female dystrophinopathy: review of current literature. Neuromuscul Disord 28:572–581. 10.1016/j.nmd.2018.04.005 29801751 10.1016/j.nmd.2018.04.005

[18] Solheim TÅ, Fornander F, Raja AA et al (2021) Cardiac involvement in women with pathogenic dystrophin gene variants. Front Neurol 12:707838. 10.3389/fneur.2021.707838 34385974 10.3389/fneur.2021.707838 PMC8353322

[19] D’Amario D, Amodeo A, Adorisio R et al (2017) A current approach to heart failure in Duchenne muscular dystrophy. Heart 103:1770–1779. 10.1136/heartjnl-2017-311269 28668906 10.1136/heartjnl-2017-311269

[20] Rafael-Fortney JA, Chadwick JA, Raman SV (2016) Duchenne muscular dystrophy mice and men: can understanding a genetic cardiomyopathy inform treatment of other myocardial diseases?? Circ Res 118:1059–1061. 10.1161/CIRCRESAHA.116.308402 27034274 10.1161/CIRCRESAHA.116.308402 PMC4819164

[21] McNally EM, Kaltman JR, Benson DW et al (2015) Contemporary cardiac issues in Duchenne muscular dystrophy. Working group of the National heart, lung, and blood Institute in collaboration with parent project muscular dystrophy. Circulation 131:1590–1598. 10.1161/CIRCULATIONAHA.114.015151 25940966 10.1161/CIRCULATIONAHA.114.015151 PMC4573596

[22] Birnkrant DJ, Bushby K, Bann CM et al (2018) Diagnosis and management of Duchenne muscular dystrophy, part 1: diagnosis, and neuromuscular, rehabilitation, endocrine, and gastrointestinal and nutritional management. Lancet Neurol 17:251–267. 10.1016/S1474-4422(18)30024-3 29395989 10.1016/S1474-4422(18)30024-3 PMC5869704

[23] Florczyk-Soluch U, Polak K, Dulak J (2021) The multifaceted view of heart problem in Duchenne muscular dystrophy. Cell Mol Life Sci 78:5447–5468. 10.1007/s00018-021-03862-2 34091693 10.1007/s00018-021-03862-2 PMC8257522

[24] Broomfield J, Hill M, Guglieri M et al (2021) Life expectancy in Duchenne muscular dystrophy: reproduced individual patient data meta-analysis. Neurology 97:e2304–e2314. 10.1212/WNL.0000000000012910 34645707 10.1212/WNL.0000000000012910 PMC8665435

[25] Mirski KT, Crawford TO (2014) Motor and cognitive delay in Duchenne muscular dystrophy: implication for early diagnosis. J Pediatr 165:1008–1010. 10.1016/j.jpeds.2014.07.006 25149498 10.1016/j.jpeds.2014.07.006

[26] von Maltzahn J, Jones AE, Parks RJ, Rudnicki MA (2013) Pax7 is critical for the normal function of satellite cells in adult skeletal muscle. Proc Natl Acad Sci U S A 110:16474–16479. 10.1073/pnas.1307680110 24065826 10.1073/pnas.1307680110 PMC3799311

[27] Dumont NA, Wang YX, von Maltzahn J et al (2015) Dystrophin expression in muscle stem cells regulates their polarity and asymmetric division. Nat Med 21:1455–1463. 10.1038/nm.3990 26569381 10.1038/nm.3990 PMC4839960

[28] Kottlors M, Kirschner J (2010) Elevated satellite cell number in Duchenne muscular dystrophy. Cell Tissue Res 340:541–548. 10.1007/s00441-010-0976-6 20467789 10.1007/s00441-010-0976-6

[29] Chang NC, Chevalier FP, Rudnicki MA (2016) Satellite cells in muscular dystrophy - lost in polarity. Trends Mol Med 22:479–496. 10.1016/j.molmed.2016.04.002 27161598 10.1016/j.molmed.2016.04.002 PMC4885782

[30] Esper ME, Brun CE, Lin AYT et al (2025) Intrinsic muscle stem cell dysfunction contributes to impaired regeneration in the Mdx mouse. J Cachexia Sarcopenia Muscle 16:e13682. 10.1002/jcsm.13682 39723578 10.1002/jcsm.13682 PMC11669949

[31] Ribeiro AF, Souza LS, Almeida CF et al (2019) Muscle satellite cells and impaired late stage regeneration in different murine models for muscular dystrophies. Sci Rep 9:11842. 10.1038/s41598-019-48156-7 31413358 10.1038/s41598-019-48156-7 PMC6694188

[32] Granet JA, Robertson R, Cusmano AA et al (2025) Muscle stem cells in Duchenne muscular dystrophy exhibit molecular impairments and altered cell fate trajectories impacting regenerative capacity. Cell Death Dis 16:437. 10.1038/s41419-025-07755-1 40473604 10.1038/s41419-025-07755-1 PMC12141486

[33] Fukada S, Morikawa D, Yamamoto Y et al (2010) Genetic background affects properties of satellite cells and Mdx phenotypes. Am J Pathol 176:2414–2424. 10.2353/ajpath.2010.090887 20304955 10.2353/ajpath.2010.090887 PMC2861106

[34] Sacco A, Mourkioti F, Tran R et al (2010) Short telomeres and stem cell exhaustion model Duchenne muscular dystrophy in mdx/mtr mice. Cell 143:1059–1071. 10.1016/j.cell.2010.11.039 21145579 10.1016/j.cell.2010.11.039 PMC3025608

[35] Blau HM, Webster C, Pavlath GK (1983) Defective myoblasts identified in Duchenne muscular dystrophy. Proc Natl Acad Sci USA 80:4856–4860 6576361 10.1073/pnas.80.15.4856 PMC384144

[36] Bronisz-Budzyńska I, Chwalenia K, Mucha O et al (2019) Mir-146a deficiency does not aggravate muscular dystrophy in Mdx mice. Skelet Muscle 9:22. 10.1186/s13395-019-0207-0 31412923 10.1186/s13395-019-0207-0 PMC6693262

[37] Bronisz-Budzyńska I, Kozakowska M, Podkalicka P et al (2020) The role of Nrf2 in acute and chronic muscle injury. Skelet Muscle 10:35. 10.1186/s13395-020-00255-0 33287890 10.1186/s13395-020-00255-0 PMC7722332

[38] Mucha O, Podkalicka P, Kaziród K et al (2021) Simvastatin does not alleviate muscle pathology in a mouse model of Duchenne muscular dystrophy. Skelet Muscle 11:21. 10.1186/s13395-021-00276-3 34479633 10.1186/s13395-021-00276-3 PMC8414747

[39] Pietraszek-Gremplewicz K, Kozakowska M, Bronisz-Budzynska I et al (2018) Heme oxygenase-1 influences satellite cells and progression of Duchenne muscular dystrophy in mice. Antioxid Redox Signal 29:128–148. 10.1089/ars.2017.7435 29669436 10.1089/ars.2017.7435

[40] Chal J, Oginuma M, Al Tanoury Z et al (2015) Differentiation of pluripotent stem cells to muscle fiber to model Duchenne muscular dystrophy. Nat Biotechnol 33:962–969. 10.1038/nbt.3297 26237517 10.1038/nbt.3297

[41] Moore TM, Lin AJ, Strumwasser AR et al (2020) Mitochondrial dysfunction is an early consequence of partial or complete dystrophin loss in Mdx mice. Front Physiol 11:690. 10.3389/fphys.2020.00690 32636760 10.3389/fphys.2020.00690 PMC7317021

[42] Andrysiak K, Machaj G, Priesmann D et al (2024) Dysregulated iron homeostasis in dystrophin-deficient cardiomyocytes: correction by gene editing and pharmacological treatment. Cardiovasc Res 120:69–81. 10.1093/cvr/cvad182 38078368 10.1093/cvr/cvad182 PMC10898935

[43] Mohiuddin M, Choi JJ, Lee NH et al (2020) Transplantation of Muscle Stem Cell Mitochondria Rejuvenates the Bioenergetic Function of Dystrophic Muscle. 2020.04.17.017822

[44] Matre PR, Mu X, Wu J et al (2019) CRISPR/Cas9-based dystrophin restoration reveals a novel role for dystrophin in bioenergetics and stress resistance of muscle progenitors. Stem Cells 37:1615–1628. 10.1002/stem.3094 31574188 10.1002/stem.3094 PMC6916636

[45] Sugihara H, Teramoto N, Nakamura K et al (2020) Cellular senescence-mediated exacerbation of Duchenne muscular dystrophy. Sci Rep 10:16385. 10.1038/s41598-020-73315-6 33046751 10.1038/s41598-020-73315-6 PMC7550355

[46] Cardone N, Taglietti V, Baratto S et al (2023) Myopathologic trajectory in Duchenne muscular dystrophy (DMD) reveals lack of regeneration due to senescence in satellite cells. Acta Neuropathol Commun 11:167. 10.1186/s40478-023-01657-z 37858263 10.1186/s40478-023-01657-z PMC10585739

[47] Biressi S, Miyabara EH, Gopinath SD et al (2014) A wnt-TGFβ2 axis induces a fibrogenic program in muscle stem cells from dystrophic mice. Sci Transl Med 6:267ra176. 10.1126/scitranslmed.3008411 25520397 10.1126/scitranslmed.3008411 PMC4350665

[48] Kodippili K, Rudnicki MA (2023) Satellite cell contribution to disease pathology in Duchenne muscular dystrophy. Front Physiol 14:1180980. 10.3389/fphys.2023.1180980 37324396 10.3389/fphys.2023.1180980 PMC10266354

[49] Filippelli RL, Chang NC (2022) Empowering muscle stem cells for the treatment of Duchenne muscular dystrophy. Cells Tissues Organs 211:641–654. 10.1159/000514305 33910206 10.1159/000514305

[50] Pradhan S, Ghosh D, Srivastava NK et al (2006) Prednisolone in Duchenne muscular dystrophy with imminent loss of ambulation. J Neurol 253:1309–1316. 10.1007/s00415-006-0212-1 16786214 10.1007/s00415-006-0212-1

[51] Henricson EK, Abresch RT, Cnaan A et al (2013) The cooperative international neuromuscular research group Duchenne natural history study: glucocorticoid treatment preserves clinically meaningful functional milestones and reduces rate of disease progression as measured by manual muscle testing and other commonly used clinical trial outcome measures. Muscle Nerve 48:55–67. 10.1002/mus.23808 23649481 10.1002/mus.23808 PMC4103170

[52] Victor RG, Sweeney HL, Finkel R et al (2017) A phase 3 randomized placebo-controlled trial of Tadalafil for Duchenne muscular dystrophy. Neurology 89:1811–1820. 10.1212/WNL.0000000000004570 28972192 10.1212/WNL.0000000000004570 PMC5664308

[53] Guiraud S, Davies KE (2017) Pharmacological advances for treatment in Duchenne muscular dystrophy. Curr Opin Pharmacol 34:36–48. 10.1016/j.coph.2017.04.002 28486179 10.1016/j.coph.2017.04.002

[54] Kourakis S, Timpani CA, Campelj DG et al (2021) Standard of care versus new-wave corticosteroids in the treatment of Duchenne muscular dystrophy: can we do better? Orphanet J Rare Dis 16:117. 10.1186/s13023-021-01758-9 33663533 10.1186/s13023-021-01758-9 PMC7934375

[55] Escolar DM, Hache LP, Clemens PR et al (2011) Randomized, blinded trial of weekend vs daily prednisone in Duchenne muscular dystrophy. Neurology 77:444–452. 10.1212/WNL.0b013e318227b164 21753160 10.1212/WNL.0b013e318227b164 PMC3146308

[56] Mah JK, Clemens PR, Guglieri M et al (2022) Efficacy and safety of Vamorolone in Duchenne muscular dystrophy: a 30-month nonrandomized controlled open-label extension trial. JAMA Netw Open 5:e2144178. 10.1001/jamanetworkopen.2021.44178 35076703 10.1001/jamanetworkopen.2021.44178 PMC8790668

[57] Myszka M, Mucha O, Podkalicka P et al (2023) Sodium hydrosulfide moderately alleviates the hallmark symptoms of Duchenne muscular dystrophy in Mdx mice. Eur J Pharmacol 175928. 10.1016/j.ejphar.2023.175928 37507045 10.1016/j.ejphar.2023.175928

[58] Mucha O, Myszka M, Podkalicka P et al (2023) Proteome profiling of the dystrophic Mdx mice diaphragm. Biomolecules 13:1648. 10.3390/biom13111648 38002330 10.3390/biom13111648 PMC10669179

[59] Kaziród K, Myszka M, Dulak J, Łoboda A (2022) Hydrogen sulfide as a therapeutic option for the treatment of Duchenne muscular dystrophy and other muscle-related diseases. Cell Mol Life Sci 79:608. 10.1007/s00018-022-04636-0 36441348 10.1007/s00018-022-04636-0 PMC9705465

[60] Łoboda A, Dulak J (2024) Cardioprotective effects of hydrogen sulfide and its potential therapeutic implications in the amelioration of Duchenne muscular dystrophy cardiomyopathy. Cells 13:158. 10.3390/cells13020158 38247849 10.3390/cells13020158 PMC10814317

[61] Myszka M, Jakubczak E, Mucha O et al (2025) GYY4137, a slow-releasing hydrogen sulfide donor, attenuates skeletal muscle abnormalities in a murine model of Duchenne muscular dystrophy. Antioxid Redox Signal. 10.1089/ars.2024.0702 40476490 10.1089/ars.2024.0702

[62] Blake DJ, Tinsley JM, Davies KE (1996) Utrophin: a structural and functional comparison to dystrophin. Brain Pathol 6:37–47. 10.1111/j.1750-3639.1996.tb00781.x 8866746 10.1111/j.1750-3639.1996.tb00781.x

[63] Clerk A, Morris GE, Dubowitz V et al (1993) Dystrophin-related protein, utrophin, in normal and dystrophic human fetal skeletal muscle. Histochem J 25:554–561. 10.1007/bf02388063 8407365

[64] Sun C, Shen L, Zhang Z, Xie X (2020) Therapeutic strategies for Duchenne muscular dystrophy: an update. Genes (Basel) 11:E837. 10.3390/genes11080837 10.3390/genes11080837 PMC7463903 32717791

[65] Fortunato F, Farnè M, Ferlini A (2021) The DMD gene and therapeutic approaches to restore dystrophin. Neuromuscul Disord 31:1013–1020. 10.1016/j.nmd.2021.08.004 34736624 10.1016/j.nmd.2021.08.004

[66] Koenig M, Hoffman EP, Bertelson CJ et al (1987) Complete cloning of the Duchenne muscular dystrophy (DMD) cDNA and preliminary genomic organization of the DMD gene in normal and affected individuals. Cell 50:509–517. 10.1016/0092-8674(87)90504-6 3607877 10.1016/0092-8674(87)90504-6

[67] Lee CC, Pearlman JA, Chamberlain JS, Caskey CT (1991) Expression of recombinant dystrophin and its localization to the cell membrane. Nature 349:334–336. 10.1038/349334a0 1824797 10.1038/349334a0

[68] Gregorevic P, Blankinship MJ, Allen JM et al (2004) Systemic delivery of genes to striated muscles using adeno-associated viral vectors. Nat Med 10:828–834. 10.1038/nm1085 15273747 10.1038/nm1085 PMC1365046

[69] Tasfaout H, Halbert CL, McMillen TS et al (2024) Split intein-mediated protein trans-splicing to express large dystrophins. Nature 632:192–200. 10.1038/s41586-024-07710-8 39020181 10.1038/s41586-024-07710-8 PMC11335042

[70] Zhou Y, Zhang C, Xiao W et al (2024) Systemic delivery of full-length dystrophin in Duchenne muscular dystrophy mice. Nat Commun 15:6141. 10.1038/s41467-024-50569-6 39034316 10.1038/s41467-024-50569-6 PMC11271493

[71] Tasfaout H, McMillen TS, Reyes TR et al (2025) Expression of full-length dystrophin reverses muscular dystrophy defects in young and old mdx4cv mice. J Clin Invest. 10.1172/JCI189075 40493400 10.1172/JCI189075 PMC12321383

[72] Tedesco FS, Dellavalle A, Diaz-Manera J et al (2010) Repairing skeletal muscle: regenerative potential of skeletal muscle stem cells. J Clin Invest 120:11–19. 10.1172/JCI40373 20051632 10.1172/JCI40373 PMC2798695

[73] Relaix F, Zammit PS (2012) Satellite cells are essential for skeletal muscle regeneration: the cell on the edge returns centre stage. Development 139:2845–2856. 10.1242/dev.069088 22833472 10.1242/dev.069088

[74] Duan D, Goemans N, Takeda S et al (2021) Duchenne muscular dystrophy. Nat Rev Dis Primers 7:1–19. 10.1038/s41572-021-00248-3 33602943 10.1038/s41572-021-00248-3 PMC10557455

[75] Cerletti M, Jurga S, Witczak CA et al (2008) Highly efficient, functional engraftment of skeletal muscle stem cells in dystrophic muscles. Cell 134:37–47. 10.1016/j.cell.2008.05.049 18614009 10.1016/j.cell.2008.05.049 PMC3665268

[76] Marg A, Escobar H, Gloy S et al (2014) Human satellite cells have regenerative capacity and are genetically manipulable. J Clin Invest 124:4257–4265. 10.1172/JCI63992 25157816 10.1172/JCI63992 PMC4191042

[77] Montarras D, Morgan J, Collins C et al (2005) Direct isolation of satellite cells for skeletal muscle regeneration. Science 309:2064–2067. 10.1126/science.1114758 16141372 10.1126/science.1114758

[78] Sacco A, Doyonnas R, Kraft P et al (2008) Self-renewal and expansion of single transplanted muscle stem cells. Nature 456:502–506. 10.1038/nature07384 18806774 10.1038/nature07384 PMC2919355

[79] Tanaka KK, Hall JK, Troy AA et al (2009) Syndecan-4-expressing muscle progenitor cells in the SP engraft as satellite cells during muscle regeneration. Cell Stem Cell 4:217–225. 10.1016/j.stem.2009.01.016 19265661 10.1016/j.stem.2009.01.016 PMC3142572

[80] Xu X, Wilschut KJ, Kouklis G et al (2015) Human satellite cell transplantation and regeneration from diverse skeletal muscles. Stem Cell Rep 5:419–434. 10.1016/j.stemcr.2015.07.016 10.1016/j.stemcr.2015.07.016 PMC4618654 26352798

[81] Negroni E, Riederer I, Chaouch S et al (2009) In vivo myogenic potential of human CD133 + muscle-derived stem cells: a quantitative study. Mol Ther 17:1771–1778. 10.1038/mt.2009.167 19623164 10.1038/mt.2009.167 PMC2835017

[82] Gilbert PM, Havenstrite KL, Magnusson KEG et al (2010) Substrate elasticity regulates skeletal muscle stem cell self-renewal in culture. Science 329:1078–1081. 10.1126/science.1191035 20647425 10.1126/science.1191035 PMC2929271

[83] Partridge TA, Grounds M, Sloper JC (1978) Evidence of fusion between host and donor myoblasts in skeletal muscle grafts. Nature 273:306–308. 10.1038/273306a0 652035 10.1038/273306a0

[84] Partridge TA, Morgan JE, Coulton GR et al (1989) Conversion of Mdx myofibres from dystrophin-negative to -positive by injection of normal myoblasts. Nature 337:176–179. 10.1038/337176a0 2643055 10.1038/337176a0

[85] Partridge TA (1991) Invited review: myoblast transfer: a possible therapy for inherited myopathies? Muscle Nerve 14:197–212. 10.1002/mus.880140302 2041542 10.1002/mus.880140302

[86] Law PK, Goodwin TG, Fang QW et al (1991) Myoblast transfer therapy for Duchenne muscular dystrophy. Acta Paediatr Jpn 33:206–215. 10.1111/j.1442-200x.1991.tb01545.x 1957647 10.1111/j.1442-200x.1991.tb01545.x

[87] Law PK, Goodwin TG, Fang Q et al (1992) Feasibility, safety, and efficacy of myoblast transfer therapy on Duchenne muscular dystrophy boys. Cell Transplant 1:235–244. 10.1177/0963689792001002-305 1344295 10.1177/0963689792001002-305

[88] Law PK, Goodwin TG, Fang Q et al (1993) Cell transplantation as an experimental treatment for Duchenne muscular dystrophy. Cell Transplant 2:485–505. 10.1177/096368979300200607 8167934 10.1177/096368979300200607

[89] Gussoni E, Blau HM, Kunkel LM (1997) The fate of individual myoblasts after transplantation into muscles of DMD patients. Nat Med 3:970–977. 10.1038/nm0997-970 9288722 10.1038/nm0997-970

[90] Mendell JR, Kissel JT, Amato AA et al (1995) Myoblast transfer in the treatment of Duchenne’s muscular dystrophy. N Engl J Med 333:832–838. 10.1056/NEJM199509283331303 7651473 10.1056/NEJM199509283331303

[91] Morandi L, Bernasconi P, Gebbia M et al (1995) Lack of mRNA and dystrophin expression in DMD patients three months after myoblast transfer. Neuromuscul Disord 5:291–295. 10.1016/0960-8966(94)00070-p 7580241 10.1016/0960-8966(94)00070-p

[92] Fischer UM, Harting MT, Jimenez F et al (2009) Pulmonary passage is a major obstacle for intravenous stem cell delivery: the pulmonary first-pass effect. Stem Cells Dev 18:683–692. 10.1089/scd.2008.0253 19099374 10.1089/scd.2008.0253 PMC3190292

[93] Skuk D, Tremblay JP (2014) First study of intra-arterial delivery of myogenic mononuclear cells to skeletal muscles in primates. Cell Transpl 23 Suppl 1:S141-150. 10.3727/096368914X685032 10.3727/096368914X685032 25303080

[94] Happi Mbakam C, Lamothe G, Tremblay JP (2022) Therapeutic strategies for dystrophin replacement in Duchenne muscular dystrophy. Front Med Lausanne 9:859930. 10.3389/fmed.2022.859930 35419381 10.3389/fmed.2022.859930 PMC8995704

[95] Saleh KK, Switzler C, Hicks MR et al (2023) Duchenne muscular dystrophy disease severity impacts skeletal muscle progenitor cells systemic delivery. Front Physiol. 10.3389/fphys.2023.1190524 37228827 10.3389/fphys.2023.1190524 PMC10203213

[96] Dellavalle A, Sampaolesi M, Tonlorenzi R et al (2007) Pericytes of human skeletal muscle are myogenic precursors distinct from satellite cells. Nat Cell Biol 9:255–267. 10.1038/ncb1542 17293855 10.1038/ncb1542

[97] Sampaolesi M, Blot S, D’Antona G et al (2006) Mesoangioblast stem cells ameliorate muscle function in dystrophic dogs. Nature 444:574–579. 10.1038/nature05282 17108972 10.1038/nature05282

[98] Tedesco FS, Hoshiya H, D’Antona G et al (2011) Stem cell-mediated transfer of a human artificial chromosome ameliorates muscular dystrophy. Sci Transl Med 3:96ra78. 10.1126/scitranslmed.3002342 21849666 10.1126/scitranslmed.3002342

[99] Lorant J, Saury C, Schleder C et al (2018) Skeletal muscle regenerative potential of human MuStem cells following transplantation into injured mice muscle. Mol Ther 26:618–633. 10.1016/j.ymthe.2017.10.013 29221805 10.1016/j.ymthe.2017.10.013 PMC5835152

[100] Cossu G, Previtali SC, Napolitano S et al (2015) Intra-arterial transplantation of HLA-matched donor mesoangioblasts in Duchenne muscular dystrophy. EMBO Mol Med 7:1513–1528. 10.15252/emmm.201505636 26543057 10.15252/emmm.201505636 PMC4693504

[101] Cossu G, Tonlorenzi R, Brunelli S et al (2022) Mesoangioblasts at 20: from the embryonic aorta to the patient bed. Front Genet 13:1056114. 10.3389/fgene.2022.1056114 36685855 10.3389/fgene.2022.1056114 PMC9845585

[102] Rouger K, Larcher T, Dubreil L et al (2011) Systemic delivery of allogenic muscle stem cells induces long-term muscle repair and clinical efficacy in Duchenne muscular dystrophy dogs. Am J Pathol 179:2501–2518. 10.1016/j.ajpath.2011.07.022 21924229 10.1016/j.ajpath.2011.07.022 PMC3204088

[103] Lorant J, Larcher T, Jaulin N et al (2018) Vascular delivery of allogeneic MuStem cells in dystrophic dogs requires only short-term immunosuppression to avoid host immunity and generate clinical/tissue benefits. Cell Transplant 27:1096–1110. 10.1177/0963689718776306 29871519 10.1177/0963689718776306 PMC6158548

[104] Charrier M, Lorant J, Contreras-Lopez R et al (2022) Human MuStem cells repress T-cell proliferation and cytotoxicity through both paracrine and contact-dependent pathways. Stem Cell Res Ther 13:7. 10.1186/s13287-021-02681-3 35012660 10.1186/s13287-021-02681-3 PMC8751303

[105] Charrier M, Leroux I, Pichon J et al (2024) Human MuStem cells are competent to fuse with nonhuman primate myofibers in a clinically relevant transplantation context: a proof-of-concept study. J Neuropathol Exp Neurol 83:684–694. 10.1093/jnen/nlae044 38752570 10.1093/jnen/nlae044

[106] Darabi R, Gehlbach K, Bachoo RM et al (2008) Functional skeletal muscle regeneration from differentiating embryonic stem cells. Nat Med 14:134–143. 10.1038/nm1705 18204461 10.1038/nm1705

[107] Incitti T, Magli A, Darabi R et al (2019) Pluripotent stem cell-derived myogenic progenitors remodel their molecular signature upon in vivo engraftment. Proc Natl Acad Sci U S A 116:4346–4351. 10.1073/pnas.1808303116 30760602 10.1073/pnas.1808303116 PMC6410870

[108] Azzag K, Magli A, Kiley J et al (2025) Preclinical quality, safety, and efficacy of a CGMP iPSC-derived myogenic progenitor product for the treatment of muscular dystrophies. Mol Ther S. 10.1016/j.ymthe.2025.07.007. 1525-0016(25)00543-X 10.1016/j.ymthe.2025.07.007 PMC12338975 40682272

[109] Shelton M, Metz J, Liu J et al (2014) Derivation and expansion of PAX7-positive muscle progenitors from human and mouse embryonic stem cells. Stem Cell Reports 3:516–529. 10.1016/j.stemcr.2014.07.001 25241748 10.1016/j.stemcr.2014.07.001 PMC4266001

[110] Sun C, Kannan S, Choi IY et al (2022) Human pluripotent stem cell-derived myogenic progenitors undergo maturation to quiescent satellite cells upon engraftment. Cell Stem Cell 29:610–619e5. 10.1016/j.stem.2022.03.004 35395188 10.1016/j.stem.2022.03.004 PMC9000524

[111] Filareto A, Parker S, Darabi R et al (2013) An ex vivo gene therapy approach to treat muscular dystrophy using inducible pluripotent stem cells. Nat Commun 4:1549. 10.1038/ncomms2550 23462992 10.1038/ncomms2550 PMC3595133

[112] Sato M, Shiba N, Miyazaki D et al (2019) Amelioration of intracellular Ca2 + regulation by exon-45 skipping in Duchenne muscular dystrophy-induced pluripotent stem cell-derived cardiomyocytes. Biochem Biophys Res Commun 520:179–185. 10.1016/j.bbrc.2019.09.095 31585729 10.1016/j.bbrc.2019.09.095

[113] Zhao M, Tazumi A, Takayama S et al (2020) Induced fetal human muscle stem cells with high therapeutic potential in a mouse muscular dystrophy model. Stem Cell Reports 15:80–94. 10.1016/j.stemcr.2020.06.004 32619494 10.1016/j.stemcr.2020.06.004 PMC7363940

[114] Kowala A, Boot J, Meng J et al (2025) Engineered human myogenic cells in hydrogels generate innervated vascularized myofibers within dystrophic mouse muscle on long-term engraftment. Cell Rep Med 6:102019. 10.1016/j.xcrm.2025.102019 40056909 10.1016/j.xcrm.2025.102019 PMC11970389

[115] Chan SS-K, Arpke RW, Filareto A et al (2018) Skeletal muscle stem cells from PSC-Derived teratomas have functional regenerative capacity. Cell Stem Cell 23:74–85e6. 10.1016/j.stem.2018.06.010 29979993 10.1016/j.stem.2018.06.010 PMC6752207

[116] Xie N, Chu SN, Azzag K et al (2021) In vitro expanded skeletal myogenic progenitors from pluripotent stem cell-derived teratomas have high engraftment capacity. Stem Cell Reports 16:2900–2912. 10.1016/j.stemcr.2021.10.014 34798067 10.1016/j.stemcr.2021.10.014 PMC8693664

[117] Wu J, Matthias N, Lo J et al (2018) A myogenic Double-Reporter human pluripotent stem cell line allows prospective isolation of skeletal muscle progenitors. Cell Rep 25:1966–1981e4. 10.1016/j.celrep.2018.10.067 30428361 10.1016/j.celrep.2018.10.067 PMC6287935

[118] Nalbandian M, Zhao M, Sasaki-Honda M et al (2021) Characterization of hiPSC-derived muscle progenitors reveals distinctive markers for myogenic cell purification toward cell therapy. Stem Cell Reports 16:883–898. 10.1016/j.stemcr.2021.03.004 33798449 10.1016/j.stemcr.2021.03.004 PMC8072070

[119] Dhoke NR, Kim H, Azzag K et al (2024) A novel CRISPR-Cas9 strategy to target DYSTROPHIN mutations downstream of exon 44 in patient-specific DMD iPSCs. Cells 13:972. 10.3390/cells13110972 38891104 10.3390/cells13110972 PMC11171783

[120] Crist SB, Azzag K, Kiley J et al (2024) The adult environment promotes the transcriptional maturation of human iPSC-derived muscle grafts. NPJ Regen Med 9:16. 10.1038/s41536-024-00360-4 38575647 10.1038/s41536-024-00360-4 PMC10994941

[121] FDA Clears Investigational New Drug application for muscular dystrophy treatment from University of Minnesota startup Myogenica - BioSpace https://www.biospace.com/fda-clears-investigational-new-drug-application-for-muscular-dystrophy-treatment-from-university-of-minnesota-startup-myogenica. Accessed 25 Apr 2025

[122] Xie N, Chu SN, Schultz CB, Chan SSK (2023) Efficient muscle regeneration by human PSC-derived CD82 + ERBB3 + NGFR + skeletal myogenic progenitors. Cells 12:362. 10.3390/cells12030362 36766703 10.3390/cells12030362 PMC9913306

[123] Alexander MS, Rozkalne A, Colletta A et al (2016) CD82 is a marker for prospective isolation of human muscle satellite cells and is linked to muscular dystrophies. Cell Stem Cell 19:800–807. 10.1016/j.stem.2016.08.006 27641304 10.1016/j.stem.2016.08.006 PMC5135584

[124] Bou Akar R, Lama C, Aubin D et al (2024) Generation of highly pure pluripotent stem cell-derived myogenic progenitor cells and myotubes. Stem Cell Reports 19:84–99. 10.1016/j.stemcr.2023.11.002 38101399 10.1016/j.stemcr.2023.11.002 PMC10828960

[125] Mashinchian O, De Franceschi F, Nassiri S et al (2022) An engineered multicellular stem cell niche for the 3d derivation of human myogenic progenitors from iPSCs. EMBO J 41:e110655. 10.15252/embj.2022110655 35703167 10.15252/embj.2022110655 PMC9289707

[126] Głowniak-Kwitek U, Caballero AL, Bronisz-Budzyńska I et al (2023) Effect of heme oxygenase-1 on the differentiation of human myoblasts and the regeneration of murine skeletal muscles after acute and chronic injury. Pharmacol Rep 75:397–410. 10.1007/s43440-023-00475-3 36918494 10.1007/s43440-023-00475-3 PMC10060298

[127] Boyer O, Butler-Browne G, Chinoy H et al (2021) Myogenic cell transplantation in genetic and acquired diseases of skeletal muscle. Front Genet 12:702547. 10.3389/fgene.2021.702547 34408774 10.3389/fgene.2021.702547 PMC8365145

[128] Choi S, Ferrari G, Moyle LA et al (2022) Assessing and enhancing migration of human myogenic progenitors using directed iPS cell differentiation and advanced tissue modelling. EMBO Mol Med 14:e14526. 10.15252/emmm.202114526 36161772 10.15252/emmm.202114526 PMC9549733

[129] Bianco P, Cao X, Frenette PS et al (2013) The meaning, the sense and the significance: translating the science of mesenchymal stem cells into medicine. Nat Med 19:35–42. 10.1038/nm.3028 23296015 10.1038/nm.3028 PMC3998103

[130] Langrzyk A, Nowak WN, Stępniewski J et al (2018) Critical view on mesenchymal stromal cells in regenerative medicine. Antioxid Redox Signal 29:169–190. 10.1089/ars.2017.7159 28874054 10.1089/ars.2017.7159

[131] Sipp D, Caulfield T, Kaye J et al (2017) Marketing of unproven stem cell-based interventions: a call to action. Sci Transl Med 9:eaag0426. 10.1126/scitranslmed.aag0426 28679655 10.1126/scitranslmed.aag0426

[132] Rodriguez A-M, Pisani D, Dechesne CA et al (2005) Transplantation of a multipotent cell population from human adipose tissue induces dystrophin expression in the immunocompetent Mdx mouse. J Exp Med 201(9):1397–1405. 10.1084/jem.20042224 15867092 10.1084/jem.20042224 PMC2213197

[133] Vieira NM, Valadares M, Zucconi E et al (2012) Human adipose-derived mesenchymal stromal cells injected systemically into GRMD dogs without immunosuppression are able to reach the host muscle and express human dystrophin. Cell Transplant 21(7):1407–1417. 10.3727/096368911X 23168016 10.3727/096368911X

[134] Sacchetti B, Funari A, Remoli C et al (2016) No identical mesenchymal stem cells at different times and sites: human committed progenitors of distinct origin and differentiation potential are incorporated as adventitial cells in microvessels. Stem Cell Reports 6:897–913. 10.1016/j.stemcr.2016.05.011 27304917 10.1016/j.stemcr.2016.05.011 PMC4912436

[135] Tang A, Yokota T (2024) Duchenne muscular dystrophy: promising early-stage clinical trials to watch. Expert Opin Investig Drugs 33:201–217. 10.1080/13543784.2024.2313105 38291016 10.1080/13543784.2024.2313105

[136] Dulak J, Pecyna M (2023) Unproven cell interventions in Poland and the exploitation of European union law on advanced therapy medicinal products. Stem Cell Rep 18:1610–1620. 10.1016/j.stemcr.2023.05.017 10.1016/j.stemcr.2023.05.017 PMC10444563 37390824

[137] Sipp D, Robey PG, Turner L (2018) Clear up this stem-cell mess. Nature 561:455–457. 10.1038/d41586-018-06756-9 30258150 10.1038/d41586-018-06756-9

[138] De Luca M, Aiuti A, Cossu G et al (2019) Advances in stem cell research and therapeutic development. Nat Cell Biol 21:801–811. 10.1038/s41556-019-0344-z 31209293 10.1038/s41556-019-0344-z

[139] Aartsma-Rus A (2023) Dystrophin Expressing Chimeric (DEC) Cell Therapy for Duchenne Muscular Dystrophy: A First-in-Human Study with Minimum 6 Months Follow-up • World Duchenne https://www.worldduchenne.org/news/apaperaday-dystrophin-expressing-chimeric-dec-cell-therapy-for-duchenne-muscular-dystrophy-a-first-in-human-study-with-minimum-6-months-follow-up/. In: World Duchene.https://www.worldduchenne.org/news/apaperaday-dystrophin-expressing-chimeric-dec-cell-therapy-for-duchenne-muscular-dystrophy-a-first-in-human-study-with-minimum-6-months-follow-up/Accessed 20 Jan 2025 10.1007/s12015-023-10530-4 PMC10366026 37000376

[140] Heydemann A, Bieganski G, Wachowiak J et al (2023) Dystrophin expressing chimeric (DEC) cell therapy for Duchenne muscular dystrophy: a first-in-human study with minimum 6 months follow-up. Stem Cell Rev Rep 19:1340–1359. 10.1007/s12015-023-10530-4 37000376 10.1007/s12015-023-10530-4 PMC10366026

[141] Siemionow M, Cwykiel J, Heydemann A et al (2018) Dystrophin expressing chimeric (DEC) human cells provide a potential therapy for Duchenne muscular dystrophy. Stem Cell Rev Rep 14:370–384. 10.1007/s12015-018-9807-z 29546607 10.1007/s12015-018-9807-z PMC5960489

[142] Siemionow M, Malik M, Langa P et al (2019) Cardiac protection after systemic transplant of dystrophin expressing chimeric (DEC) cells to the Mdx mouse model of Duchenne muscular dystrophy. Stem Cell Rev Rep 15:827–841. 10.1007/s12015-019-09916-0 31612351 10.1007/s12015-019-09916-0 PMC6925071

[143] Galvez BG, Sampaolesi M, Barbuti A et al (2008) Cardiac mesoangioblasts are committed, self-renewable progenitors, associated with small vessels of juvenile mouse ventricle. Cell Death Differ 15:1417–1428. 10.1038/cdd.2008.75 18497758 10.1038/cdd.2008.75

[144] Chun JL, O’Brien R, Song MH et al (2013) Injection of vessel-derived stem cells prevents dilated cardiomyopathy and promotes angiogenesis and endogenous cardiac stem cell proliferation in Mdx/utrn-/- but not aged Mdx mouse models for Duchenne muscular dystrophy. Stem Cells Transl Med 2:68–80. 10.5966/sctm.2012-0107 23283493 10.5966/sctm.2012-0107 PMC3659745

[145] Aminzadeh MA, Rogers RG, Fournier M et al (2018) Exosome-mediated benefits of cell therapy in mouse and human models of Duchenne muscular dystrophy. Stem Cell Reports 10:942–955. 10.1016/j.stemcr.2018.01.023 29478899 10.1016/j.stemcr.2018.01.023 PMC5918344

[146] Rogers RG, Fournier M, Sanchez L et al (2019) Disease-modifying bioactivity of intravenous cardiosphere-derived cells and exosomes in Mdx mice. JCI Insight 4:e125754. 10.1172/jci.insight.125754 30944252 10.1172/jci.insight.125754 PMC6483717

[147] Rogers RG, Antich J, Fournier M et al (2025) Long-term preservation of muscle function and structure by repeated administration of cardiosphere-derived cells in Mdx mice. Stem Cell Reports 20:102468. 10.1016/j.stemcr.2025.102468 40118057 10.1016/j.stemcr.2025.102468 PMC12069882

[148] Taylor M, Jefferies J, Byrne B et al (2019) Cardiac and skeletal muscle effects in the randomized HOPE-Duchenne trial. Neurology 92:e866–e878. 10.1212/WNL.0000000000006950 30674601 10.1212/WNL.0000000000006950 PMC6396968

[149] McDonald CM, Marbán E, Hendrix S et al (2022) Repeated intravenous cardiosphere-derived cell therapy in late-stage Duchenne muscular dystrophy (HOPE-2): a multicentre, randomised, double-blind, placebo-controlled, phase 2 trial. Lancet 399:1049–1058. 10.1016/S0140-6736(22)00012-5 35279258 10.1016/S0140-6736(22)00012-5

[150] Davis DR (2022) Cell therapy for patients with Duchenne muscular dystrophy. Lancet 399:1024–1025. 10.1016/S0140-6736(22)00185-4 35279246 10.1016/S0140-6736(22)00185-4

[151] Capricor T (2024) Capricor Therapeutics Announces Positive Long-Term Data from HOPE-2 OLE Study in Duchenne Muscular Dystrophy at 2024 World Muscle Society Congress. In: Capricor Therapeutics, Inc. https://www.capricor.com/investors/news-events/press-releases/detail/294/capricor-therapeutics-announces-positive-long-term-data. Accessed 6 Mar 2025

[152] Manalac T (2025) Capricor Plunges on FDA Rejection of DMD Cell Therapy. In: BioSpace. https://www.biospace.com/fda/capricor-plunges-on-fda-rejection-of-dmd-cell-therapy. Accessed 21 Aug 2025

[153] Jebran A-F, Seidler T, Tiburcy M et al (2025) Engineered heart muscle allografts for heart repair in primates and humans. Nature. 10.1038/s41586-024-08463-0 39880949 10.1038/s41586-024-08463-0 PMC11903342

[154] Karbassi E, Fenix A, Marchiano S et al (2020) Cardiomyocyte maturation: advances in knowledge and implications for regenerative medicine. Nat Rev Cardiol 17:341–359. 10.1038/s41569-019-0331-x 32015528 10.1038/s41569-019-0331-x PMC7239749

[155] Chien KR, Frisén J, Fritsche-Danielson R et al (2019) Regenerating the field of cardiovascular cell therapy. Nat Biotechnol 37:232–237. 10.1038/s41587-019-0042-1 30778231 10.1038/s41587-019-0042-1

[156] Murry CE, MacLellan WR (2020) Stem cells and the heart-the road ahead. Science 367:854–855. 10.1126/science.aaz3650 32079761 10.1126/science.aaz3650

[157] Dulak J, Zieliński T, Józkowicz A, Łoboda A (2025) Pluripotent stem cell-based approaches for heart repair and the potential of genetic modifications. Mol Ther S. 10.1016/j.ymthe.2025.07.009. 1525-0016(25)00545–3 10.1016/j.ymthe.2025.07.009 PMC12848174 40682270

[158] Kim H, Perlingeiro RCR (2022) Generation of human myogenic progenitors from pluripotent stem cells for in vivo regeneration. Cell Mol Life Sci 79:406. 10.1007/s00018-022-04434-8 35802202 10.1007/s00018-022-04434-8 PMC9270264

[159] Zambon AA, Falzone YM, Bolino A, Previtali SC (2024) Molecular mechanisms and therapeutic strategies for neuromuscular diseases. Cell Mol Life Sci 81:198. 10.1007/s00018-024-05229-9 38678519 10.1007/s00018-024-05229-9 PMC11056344

[160] Selvaraj S, Dhoke NR, Kiley J et al (2019) Gene correction of LGMD2A patient-specific iPSCs for the development of targeted autologous cell therapy. Mol Ther 27:2147–2157. 10.1016/j.ymthe.2019.08.011 31501033 10.1016/j.ymthe.2019.08.011 PMC6904833

[161] Müthel S, Marg A, Ignak B et al (2023) Cas9-induced single cut enables highly efficient and template-free repair of a muscular dystrophy causing founder mutation. Mol Ther 31:494–511. 10.1016/j.omtn.2023.02.005 10.1016/j.omtn.2023.02.005 PMC9972404 36865086

[162] Levy J (2024) Vita Therapeutics letter to the LGMD2A/R1 community. In: Coalition to Cure Calpain 3. https://www.curecalpain3.org/vita-therapeutics-letter-to-the-lgmd2a-r1-community/. Accessed 21 Aug 2025

[163] Azzag K, Bosnakovski D, Tungtur S et al (2022) Transplantation of PSC-derived myogenic progenitors counteracts disease phenotypes in FSHD mice. NPJ Regen Med 7:43. 10.1038/s41536-022-00249-0 36056021 10.1038/s41536-022-00249-0 PMC9440030

[164] Périé S, Trollet C, Mouly V et al (2014) Autologous myoblast transplantation for oculopharyngeal muscular dystrophy: a phase i/iia clinical study. Mol Ther 22:219–225. 10.1038/mt.2013.155 23831596 10.1038/mt.2013.155 PMC3978797

[165] Rurik JG, Tombácz I, Yadegari A et al (2022) CAR T cells produced in vivo to treat cardiac injury. Science 375:91–96. 10.1126/science.abm0594 34990237 10.1126/science.abm0594 PMC9983611

[166] Aghajanian H, Kimura T, Rurik JG et al (2019) Targeting cardiac fibrosis with engineered T cells. Nature 573:430–433. 10.1038/s41586-019-1546-z 31511695 10.1038/s41586-019-1546-z PMC6752964

[167] Ferrer-Curriu G, Soler-Botija C, Charvatova S et al (2023) Preclinical scenario of targeting myocardial fibrosis with chimeric antigen receptor (CAR) immunotherapy. Biomed Pharmacother 158:114061. 10.1016/j.biopha.2022.114061 36495661 10.1016/j.biopha.2022.114061

[168] Shahvali S, Rahiman N, Jaafari MR, Arabi L (2023) Targeting fibroblast activation protein (FAP): advances in CAR-T cell, antibody, and vaccine in cancer immunotherapy. Drug Deliv Transl Res 13:2041–2056. 10.1007/s13346-023-01308-9 36840906 10.1007/s13346-023-01308-9

[169] Marigny C, Revet G, Berger A et al (2023) Abstract 13851: CAR-T cells: a potential novel strategy for mitigating fibrosis in Duchenne muscular dystrophy. Circulation 148:A13851–A13851. 10.1161/circ.148.suppl_1.13851

[170] Nelson MD, Rader F, Tang X et al (2014) Pde5 inhibition alleviates functional muscle ischemia in boys with Duchenne muscular dystrophy. Neurology 82:2085–2091. 10.1212/WNL.0000000000000498 24808022 10.1212/WNL.0000000000000498 PMC4118495

[171] Dikalov SI, Nazarewicz RR (2013) Angiotensin II-induced production of mitochondrial reactive oxygen species: potential mechanisms and relevance for cardiovascular disease. Antioxid Redox Signal 19:1085–1094. 10.1089/ars.2012.4604 22443458 10.1089/ars.2012.4604 PMC3771548

[172] Kamdar F, Garry DJ (2016) Dystrophin-deficient cardiomyopathy. J Am Coll Cardiol 67:2533–2546. 10.1016/j.jacc.2016.02.081 27230049 10.1016/j.jacc.2016.02.081

[173] Duboc D, Meune C, Pierre B et al (2007) Perindopril preventive treatment on mortality in Duchenne muscular dystrophy: 10 years’ follow-up. Am Heart J 154:596–602. 10.1016/j.ahj.2007.05.014 17719312 10.1016/j.ahj.2007.05.014

[174] Allen HD, Flanigan KM, Thrush PT et al (2013) A randomized, double-blind trial of Lisinopril and Losartan for the treatment of cardiomyopathy in Duchenne muscular dystrophy. PLoS Curr 5:ecurrents.md.2cc69a1dae4be7dfe2bcb420024ea865 10.1371/currents.md.2cc69a1dae4be7dfe2bcb420024ea865 10.1371/currents.md.2cc69a1dae4be7dfe2bcb420024ea865 PMC3871420 24459612

[175] Bangalore S, Fakheri R, Toklu B et al (2016) Angiotensin-converting enzyme inhibitors or angiotensin receptor blockers in patients without heart failure?? Insights from 254,301 patients from randomized trials. Mayo Clin Proc 91:51–60. 10.1016/j.mayocp.2015.10.019 26763511 10.1016/j.mayocp.2015.10.019

[176] Kajimoto H, Ishigaki K, Okumura K et al (2006) Beta-blocker therapy for cardiac dysfunction in patients with muscular dystrophy. Circ J 70:991–994. 10.1253/circj.70.991 16864930 10.1253/circj.70.991

[177] Viollet L, Thrush PT, Flanigan KM et al (2012) Effects of angiotensin-converting enzyme inhibitors and/or beta blockers on the cardiomyopathy in Duchenne muscular dystrophy. Am J Cardiol 110:98–102. 10.1016/j.amjcard.2012.02.064 22463839 10.1016/j.amjcard.2012.02.064

[178] Bourke JP, Watson G, Muntoni F et al (2018) Randomised placebo-controlled trial of combination ACE inhibitor and beta-blocker therapy to prevent cardiomyopathy in children with Duchenne muscular dystrophy? (DMD heart protection study): a protocol study. BMJ Open. 10.1136/bmjopen-2018-022572 30573480 10.1136/bmjopen-2018-022572 PMC6303652

[179] Ploutz M, Moore R, Ashiki M et al (2017) Spironolactone therapy for cardiomyopathy in Duchenne muscular dystrophy. J Am Coll Cardiol 69:870–870. 10.1016/S0735-1097(17)34259-6

[180] Raman SV, Hor KN, Mazur W et al (2015) Eplerenone for early cardiomyopathy in Duchenne muscular dystrophy: a randomised, double-blind, placebo-controlled trial. Lancet Neurol 14:153–161. 10.1016/S1474-4422(14)70318-7 25554404 10.1016/S1474-4422(14)70318-7 PMC4361281

[181] Raman SV, Hor KN, Mazur W et al (2017) Eplerenone for early cardiomyopathy in Duchenne muscular dystrophy: results of a two-year open-label extension trial. Orphanet J Rare Dis 12:39. 10.1186/s13023-017-0590-8 28219442 10.1186/s13023-017-0590-8 PMC5319045

[182] Chancellor DR, Davies KE, De Moor O et al (2011) Discovery of 2-arylbenzoxazoles as upregulators of utrophin production for the treatment of Duchenne muscular dystrophy. J Med Chem 54:3241–3250. 10.1021/jm200135z 21456623 10.1021/jm200135z

[183] Soblechero-Martín P, López-Martínez A, de la Puente-Ovejero L et al (2021) Utrophin modulator drugs as potential therapies for Duchenne and Becker muscular dystrophies. Neuropathol Appl Neurobiol 47:711–723. 10.1111/nan.12735 33999469 10.1111/nan.12735 PMC8518368

[184] Mozzetta C, Sartorelli V, Steinkuhler C, Puri PL (2024) HDAC inhibitors as pharmacological treatment for Duchenne muscular dystrophy: a discovery journey from bench to patients. Trends Mol Med 30:278–294. 10.1016/j.molmed.2024.01.007 38408879 10.1016/j.molmed.2024.01.007 PMC11095976

[185] Ellwood RA, Hewitt JE, Torregrossa R et al (2021) Mitochondrial hydrogen sulfide supplementation improves health in the *C. elegans* Duchenne muscular dystrophy model. Proc Natl Acad Sci U S A 118:e2018342118. 10.1073/pnas.2018342118 33627403 10.1073/pnas.2018342118 PMC7936346

[186] Panza E, Vellecco V, Iannotti FA et al (2021) Duchenne’s muscular dystrophy involves a defective transsulfuration pathway activity. Redox Biol 45:102040. 10.1016/j.redox.2021.102040 34174560 10.1016/j.redox.2021.102040 PMC8246642

[187] Saclier M, Ben Larbi S, My Ly H et al (2021) Interplay between myofibers and pro-inflammatory macrophages controls muscle damage in Mdx mice. J Cell Sci 134:jcs258429. 10.1242/jcs.258429 34471933 10.1242/jcs.258429

[188] Goudenege S, Lebel C, Huot NB et al (2012) Myoblasts derived from normal hESCs and dystrophic HiPSCs efficiently fuse with existing muscle fibers following transplantation. Mol Ther 20:2153–2167. 10.1038/mt.2012.188 22990676 10.1038/mt.2012.188 PMC3498803

[189] Darabi R, Arpke RW, Irion S et al (2012) Human ES- and iPS-derived myogenic progenitors restore DYSTROPHIN and improve contractility upon transplantation in dystrophic mice. Cell Stem Cell 10:610–619. 10.1016/j.stem.2012.02.015 22560081 10.1016/j.stem.2012.02.015 PMC3348507

[190] Hicks MR, Hiserodt J, Paras K et al (2018) ERBB3 and NGFR mark a distinct skeletal muscle progenitor cell in human development and hPSCs. Nat Cell Biol 20:46–57. 10.1038/s41556-017-0010-2 29255171 10.1038/s41556-017-0010-2 PMC5962356

[191] Young CS, Hicks MR, Ermolova NV et al (2016) A single CRISPR-Cas9 deletion strategy that targets the majority of DMD patients restores dystrophin function in hiPSC-derived muscle cells. Cell Stem Cell 18:533–540. 10.1016/j.stem.2016.01.021 26877224 10.1016/j.stem.2016.01.021 PMC4826286

[192] Choi IY, Lim H, Estrellas K et al (2016) Concordant but varied phenotypes among Duchenne muscular dystrophy patient-specific myoblasts derived using a human iPSC-based model. Cell Rep 15:2301–2312. 10.1016/j.celrep.2016.05.016 27239027 10.1016/j.celrep.2016.05.016

[193] Magli A, Incitti T, Kiley J et al (2017) PAX7 targets, CD54, integrin α9β1, and SDC2, allow isolation of human ESC/iPSC-derived myogenic progenitors. Cell Rep 19:2867–2877. 10.1016/j.celrep.2017.06.005 28658631 10.1016/j.celrep.2017.06.005 PMC5528177

[194] Kim H, Selvaraj S, Kiley J et al (2021) Genomic safe harbor expression of PAX7 for the generation of engraftable myogenic progenitors. Stem Cell Rep 16:10–19. 10.1016/j.stemcr.2020.11.001 10.1016/j.stemcr.2020.11.001 PMC7815936 33275879

[195] Frattini P, Villa C, De Santis F et al (2017) Autologous intramuscular transplantation of engineered satellite cells induces exosome-mediated systemic expression of Fukutin-related protein and rescues disease phenotype in a murine model of limb-girdle muscular dystrophy type 2I. Hum Mol Genet 26:3682–3698. 10.1093/hmg/ddx252 28666318 10.1093/hmg/ddx252 PMC5886111

[196] Azzag K, Ortiz-Cordero C, Oliveira NAJ et al (2020) Efficient engraftment of pluripotent stem cell-derived myogenic progenitors in a novel immunodeficient mouse model of limb girdle muscular dystrophy 2I. Skelet Muscle 10:10. 10.1186/s13395-020-00228-3 32321586 10.1186/s13395-020-00228-3 PMC7175515

[197] Dhoke NR, Kim H, Selvaraj S et al (2021) A universal gene correction approach for FKRP-associated dystroglycanopathies to enable autologous cell therapy. Cell Rep 36:109360. 10.1016/j.celrep.2021.109360 34260922 10.1016/j.celrep.2021.109360 PMC8327854

[198] Tedesco FS, Gerli MFM, Perani L et al (2012) Transplantation of genetically corrected human iPSC-derived progenitors in mice with limb-girdle muscular dystrophy. Sci Transl Med 4:140ra89. 10.1126/scitranslmed.3003541 22745439 10.1126/scitranslmed.3003541

[199] Ortiz-Vitali JL, Wu J, Xu N et al (2023) Disease modeling and gene correction of LGMDR21 iPSCs elucidates the role of POGLUT1 in skeletal muscle maintenance, regeneration, and the satellite cell niche. Mol Ther 33:683–697. 10.1016/j.omtn.2023.07.037 10.1016/j.omtn.2023.07.037 PMC10462830 37650119

[200] Escobar H, Di Francescantonio S, Smirnova J et al (2025) Gene-editing in patient and humanized-mice primary muscle stem cells rescues dysferlin expression in dysferlin-deficient muscular dystrophy. Nat Commun 16:120. 10.1038/s41467-024-55086-0 39747848 10.1038/s41467-024-55086-0 PMC11695731

[201] Leriche-Guérin K, Anderson LVB, Wrogemann K et al (2002) Dysferlin expression after normal myoblast transplantation in SCID and in SJL mice. Neuromuscul Disord 12:167–173. 10.1016/s0960-8966(01)00254-1 11738359 10.1016/s0960-8966(01)00254-1

[202] Darabi R, Baik J, Clee M et al (2009) Engraftment of embryonic stem cell-derived myogenic progenitors in a dominant model of muscular dystrophy. Exp Neurol 220:212–216. 10.1016/j.expneurol.2009.08.002 19682990 10.1016/j.expneurol.2009.08.002 PMC2761496

[203] Straub V, Murphy A, Udd B et al (2018) 229th ENMC international workshop: limb girdle muscular dystrophies – nomenclature and reformed classification naarden, the netherlands, 17–19 march 2017. Neuromuscul Disord 28:702–710. 10.1016/j.nmd.2018.05.007 30055862 10.1016/j.nmd.2018.05.007

